# Part 1: A Sector-Wide Survey of UK/British Isles Shelter Organisations Caring for Cats: Caregiver-Reported Approaches to Housing, Husbandry and General Care Provision

**DOI:** 10.3390/vetsci13060587

**Published:** 2026-06-16

**Authors:** Lauren R. Finka, Ana M. Barcelos, James Waterman, Avni Bhatia, Jenni L. McDonald, Rae Foreman-Worsley, Beth Skillings

**Affiliations:** 1 Feline Welfare Research Team, Cats Protection, National Cat Centre, Haywards Heath RH17 7TT, UKjames.waterman@cats.org.uk (J.W.); avni.bhatia@cats.org.uk (A.B.); jennifer.mcdonald@cats.org.uk (J.L.M.); rae.foreman-worsley@cats.org.uk (R.F.-W.);; 2 RedPony Analytics, Caernarfon LL55 4EP, UK; 3 Birkbeck College, University of London, London WC1E 7HX, UK

**Keywords:** welfare, rescue, rehoming, domestic cat, Felis catus, unowned cats, stray cats, feral cats, capacity for care

## Abstract

Meeting the physiological and psychological needs of shelter cats through appropriate care is critical to reducing stress and disease risk, as well as enabling positive homing outcomes. Shelter organisations across the British Isles provide care for many cats; however, little is known about the types of housing and husbandry approaches applied. This study, therefore, aimed to quantify current approaches to cat housing, husbandry, and general care practices, in addition to providing information relevant to local site capacity, considering reported practices against sector minimum standards where applicable. Nine hundred and sixty-one shelter organisations and/or sites caring for cats were identified and invited to complete an online survey including predominantly multiple-choice questions. A total of 393 unique responses were collected from employees and volunteers, and quantitative data were summarised descriptively. In most cases, the results provided evidence of majority alignment with sector standards, although substantial variations in reported practices were also consistently captured. While most responses described approaches supportive of meeting cats’ basic physiological needs (e.g., access to veterinary care and basic resources), psychological needs were addressed less consistently (e.g., general housing and husbandry approaches), potentially leading to poor welfare outcomes. Identified opportunities to better meet cats’ needs include more cat-friendly, low-stress approaches to pen cleaning and cat handling; greater and more consistent provisioning of within-pen resources; and improved approaches to multi-cat housing and associated decision-making. Additional opportunities to enhance both cat and human wellbeing include more structured intake and assessment processes and capacity management to support optimal cat-to-staff ratios, staff working hours, cat lengths of stay and more consistent access to isolation and emergency intake facilities.

## Simple Summary

Meeting the physiological and psychological needs of shelter cats through appropriate care is critical to reducing stress and disease risk, as well as enabling positive homing outcomes. Shelter organisations across the British Isles provide care for many cats; however, little is known about the types of housing and husbandry approaches to which they are exposed. This study identified 961 individual shelter organisations or sites actively caring for cats and invited them to complete an online survey, with 393 unique responses collected from employees and volunteers. Respondents provided information on cat housing, husbandry and general care practices, in addition to information relevant to local site capacity and operations. The results indicated that most responses described approaches supportive of meeting cats’ physiological needs (e.g., access to veterinary care and basic resources), while psychological needs were addressed less consistently (e.g., cat-friendly approaches to cleaning, handling, multi-cat housing and pen provisioning), potentially leading to poor welfare outcomes. Opportunities to more consistently meet cats’ psychological needs, in addition to those aimed at enhancing both cat and human wellbeing via capacity for care considerations, were identified.

## 1. Introduction

Animal shelters are establishments that typically provide care for unwanted and/or unowned cats and dogs. This umbrella term may include establishments otherwise referred to as (re)homing centres, rescues, sanctuaries, or foster networks. In the UK, shelters take responsibility for the housing and homing of substantial numbers of cats, with recent estimates suggesting that over 27,000 cats reside in UK shelters at any given time [1]. Shelter populations are typically transitory, with the average time in care estimated at 45 days [1,2,3]; however, many cats may live in shelters for much longer and, in some cases, indefinitely [2,3]. In general, the housing and husbandry practices to which shelter cats are exposed, the types of medical and behavioural interventions they receive, and the decisions made concerning their management and homing outcomes can all have profound impacts on their wellbeing. For example, inconsistent human interactions [4,5,6,7], social housing challenges [8] and inadequate environmental provisions [4,9,10] can all negatively impact on their physical and emotional wellbeing. Shelter environments are also notorious breeding and transmission grounds for infectious disease [11,12], potentially impacting not just physical but also psychological health [13]. Disease risk is also known to increase with length of stay and to be exacerbated by inadequate stress management, cleaning and biosecurity protocols [14,15,16,17,18].

Many shelters may operate at or over capacity, where the number of resident cats exceed the available resources required to manage populations efficiently and provide good levels of cat welfare [3,19,20]. Some shelters may also operate more like sanctuaries [21,22], where little active homing is undertaken, particularly for cats that are perceived to be ‘unhomeable’ and/or where there has been little or no homing interest from the public. In extreme cases such as hoarding, cats may remain indefinitely in over-crowded shelter conditions without adequate nutrition, sanitation or veterinary care to meet their basic needs [23,24]. Finally, many cats that end up in shelters may not be sufficiently socialised to close human proximity and physical contact or to domestic lifestyles in general [25,26,27]. Decisions to bring these cats into shelter care for more than very brief periods (e.g., to trap–neuter–return/relocate (TNR/R)) may, therefore, translate into particularly poor welfare experiences. Situations where these cats remain within shelters indefinitely or are subsequently homed as human companions could also lead to a long-term poor quality of life [28,29,30,31,32,33].

Despite the numerous ways in which the care of shelter cats and associated decision-making may impact on their health and wellbeing, the majority of the British Isles shelter sector is not currently subject to regulation or licencing. Minimum welfare and operational standards for shelter/rescue organisations are set out by the animal welfare charity Association of Dogs and Cats Homes (ADCH) [34]. Their aims are to safeguard animal welfare, support the reputation of shelters, help improve and maintain good practice in shelter animal welfare and, additionally, prepare for future licencing of the sector (should this be implemented). However, the ADCH’s minimum standards are not legislated and ADCH membership is non-compulsory. Although membership coverage across individual brick and mortar sites is unknown, uptake is likely to be incomplete. There are currently only 170 shelter organisations/individual sites listed as members across both dog and cat organisations within the British Isles, but there are thought to be approximately 1000 sites/foster networks in existence, considering only those that house cats; see McDonald et al. [1], Stavisky et al. [35] and further in the Materials and Methods Section.

A lack of legislation and its appropriate enforcement to drive the standards necessary to adequately meet the needs of shelter cats has likely facilitated a sector with diverse cat care practices. There is also no specification regarding the types of knowledge or expertise required of those caring for shelter populations, nor any formally accredited training courses or protected role titles (e.g., as opposed to ‘veterinary surgeon’). The shelter sector is also experiencing adversity in the aftermath of a challenging economic situation and associated ‘cost of living crisis’ [36]. This is in addition to a trend towards increased online purchasing and away from shelter adoption [37]. These factors have likely led to destabilising economic situations for many shelter organisations at a time where there is greater demand for their services, such as pet behavioural support and relinquishment, subsidised pet food, microchipping, neutering and other veterinary costs [38,39,40,41]. Stakeholder wellbeing is also critical to the sustainability of good-quality shelter care, and there are substantial concerns about compassion fatigue and burnout amongst animal carers [42,43].

In a shelter context, ‘best-practice’ cat care can be defined as approaches that provide suitable environments that meet the core physiological and psychological needs of cat populations. These are potentially achieved via appropriate social or single housing based on cats’ individual requirements [8,44], adequate resource provisions and enclosure design [45,46,47], preventative veterinary health care and appropriate biosecurity, handling and husbandry practices to reduce disease risk but also stress [4,11]. These approaches are likely to be best supported where sites operate at an appropriate capacity based on available housing, staff resources and where shorter lengths of stay are prioritised [11,20]. Given the sector’s challenges and limited standardisation, it is important to establish a baseline of current cat care practices and their variation in order to identify where future interventions could support consistent application of best-practice approaches.

To our knowledge, the last sector-wide surveys of UK shelters were undertaken over a decade ago [35,48,49]; these were mixed-species surveys and were not targeted to explore cat care provision in detail. To fill these knowledge gaps, this study applied a mixed methods approach to survey and report on current cat care practices across the British Isles shelter sector and to consider practices in relation to ADCH guidance [34] where relevant and available. Due to the breadth and volume of information contained within the survey responses, the current manuscript (Part 1) addresses areas relevant to meeting cats’ basic needs and in the context of local capacity and operations, with Part 2 by Finka et al. [50] (in submission) addressing behaviour assessments, management and homing decisions. Parts 3 and 4 (not yet submitted) address cat socialisation and welfare assessment. (The data collected and analysed prioritises individual site-level rather than organisation-level reporting in order to provide a realistic picture of the types of local practices and environments to which individual cats would be exposed.)

## 2. Materials and Methods

### 2.1. Ethical Approval, GDPR and Respondent Consent

The project was assessed under Nottingham Trent University (NTU) Animal Rural and Environmental Sciences (ARES) ethical protocols and classified as low risk (reference number: ARE2223M0404). All personal information provided by survey respondents was stored in a manner consistent with current General Data Protection Regulation (GDPR) (see Privacy policy | Cats Protection). The survey was hosted on a platform owned and managed by Basis Research (a fully GDPR-compliant research and insight consultancy). Respondents were not asked to disclose their name or the name of the shelter organisation they worked or volunteered for, and all survey data were fully anonymised (i.e., by removing email addresses provided by participants from their survey responses) by Basis© prior to being shared with the Cats Protection research team. At the start of the survey, respondents were informed about the purpose of the study, eligibility criteria, ethical approval, way that their data would be handled and processed, that their participation was voluntary, and that they could withdraw at any time up until the point when their data were anonymised by Basis©. Respondents were also asked to tick various boxes confirming their explicit consent to their data being used for research purposes and confirming that they understood the terms of the study.

### 2.2. Survey Questions

Following initial survey development, iterative rounds of piloting involving approximately 60 employees and volunteers across the cat rehoming and welfare sector were carried out. Rather than formal psychometric validation, feedback was sought on item clarity and face and content validity, and changes to the wording, content and survey structure were made accordingly. A total of 81 main survey questions were posed, comprising predominantly multiple-choice answer options. In some cases, answers prompted follow-on questions or open-ended text-box options to appear, enabling further detail or context to be added.

Questions were divided into 16 sections with the first few capturing basic demographic information about respondents and the nature and structure of the organisation they worked and/or volunteered for. The other sections focused on intake, housing, husbandry and general care provision (reported on in the current manuscript), approaches to cat behaviour assessments, homing decision-making and outcomes (reported on in [50]), in addition to cat socialisation and welfare assessments (to be addressed in future publications). To reduce responder fatigue, respondents were able to save their progress and return later. Respondents were also able to skip whole sections of the survey they felt were not relevant to their role. ‘Unsure/don’t know’ answer options were provided for most questions so that respondents were not forced to provide bogus answers to progress (i.e., in situations where they may not know the appropriate answer to a question). Full wording of all questions, including response options, routing logic and sections that could be skipped are available in the Supplementary Materials (Document S1: Survey Questions).

### 2.3. Survey Eligibility Criteria

To support respondent anonymity and respondents’ ability to answer questions openly and honestly, respondents were not asked to disclose the organisation/specific site within an organisation at which they worked or volunteered. Multiple responses from different individuals within the same shelter organisation were encouraged to enhance response rates. Replication and/or over representation of specific shelter organisations was not a major concern because responses to the majority of questions related to aspects of cat care and decision-making with the potential to vary substantially at the local level (either among individual cat caregivers at the same site and/or among different sites), even within the same organisation. Where respondents cared for cats across multiple shelter organisations/sites, they were instructed to answer on behalf of the organisation/site where they spent the most time. Respondents were asked to confirm they met the following eligibility criteria:

- Currently work or volunteer with an animal rehoming/rescue/shelter/sanctuary organisation within the British Isles, either full- or part-time. This includes anyone that might run their own rehoming/rescue/shelter/sanctuary organisation;
- Be directly involved in the regular provision of care to cats (other than those they owned) that are being housed by or on behalf of the above organisation. Care provided may include routine housing and husbandry and/or behaviour and welfare assessments and cat welfare support;
- Be aged 18 years or over.

### 2.4. Sampling Approach

The survey was open from 16 May to 1 October 2023. It was launched at the ADCH 2023 annual conference; launch efforts included a dedicated information stall and survey flyers placed in delegate gift bags. Subsequent targeted sampling of 961 identified shelter organisations/sites was then undertaken via the following methods:

Targeted sampling within Cats Protection (*n* = 189 contacts), as follows:

Team leaders of frontline staff caring for cats (*n* = 189) were emailed directly about the survey in July 2023 and asked to complete and/or cascade it through their internal and external shelter networks. Information about the survey was also shared via Cats Protection’s internal communications channels to extend its reach to all Cats Protection employees and volunteers directly caring for cats.

Targeted sampling across the sector (*n* = 772 contacts), as follows:

All other identifiable British Isles shelter organisations were contacted individually between June and July 2023 and asked to share the survey through their networks. Non-Cats Protection cat shelter organisations/individual sites within a larger organisation and their respective email/phone contact details were identified and extracted using a combination of searches on Cat Chat (https://www.catchat.org/index.php/cat-rescue-centres-uk-ireland) and the respective charity commission databases; Scottish charity regulator (www.oscr.org.uk), Charity commission for England and Wales (www.gov.uk/government/organisations/charity-commission), the charities regulator for Ireland (www.charitiesregulator.ie) and the Charity commission for Northern Ireland (www.charitycommissionni.org.uk); all sites were accessed on 2 June 2023. A total of 883 (non-Cats Protection) shelter organisations/individual sites were initially identified, with a final total of 772 separate organisations/individual sites within an organisation subsequently identified as currently caring for cats, for which unique up-to-date contact details could be found, and the sites successfully contacted (via email or telephone). This reduced figure was because some individual sites within an organisation could not be directly contacted as they all provided the same generic/central contact information; several organisations only had a Facebook page with no email address/phone number; several reported that they did not house/no longer housed cats; and several organisations were reportedly closed or did not provide a working email account or phone number (and so presumably may have closed) or had a full mailbox, did not answer when called, and did not provide an option to leave a message. Other methods to recruit participants during the survey period included a webinar delivered to ADCH members and newsletters distributed by ADCH, Association of Charity Vets, International Cat Care and the Veterinary Times.

To incentivise survey engagement, upon survey completion respondents were offered the opportunity to opt in to a prize draw for the chance to win a GBP 20 voucher for a popular pet accessory store, in addition to a range of online courses provided by International Cat Care and an ADCH 2024 conference place.

### 2.5. Data Analysis

Quantitative data analysis: Excel [51] was used to generate summary statistics. Tables and figures were generated using R Statistical Software 4.5.1 [52,53,54,55,56]. As multiple responses per site/organisation were permitted but data were anonymised, descriptive statistics focused on respondent-level reporting rather than clustering via site/organisation. Analyses were performed on available cases per question with no imputation. For each question item with multiple response options (i.e., a frequency rating ranging from ‘always’ to ‘never’), participants could only select one response option and percentages were calculated as a function of how many participants selected a particular option. Outliers (or potentially erroneous responses) were removed prior to analysis where appropriate, see the Results Section for specific details of where this was undertaken. Data were compared against ADCH minimum standards/recommendations [34] where these were relevant and available. In order to keep the data analysis and reporting to a reasonable length and to avoid non-robust comparisons among subgroups, responses were analysed as a whole rather than as a function of ADCH membership/non-membership or organisation size/structure.

## 3. Results

Full results are included in the Supplementary Materials (Document S2: Survey Results), with values reported selectively in the main text to reduce the study’s length and optimise question interpretability. Data are summarised and structured relative to the study’s aims; current cat care approaches relevant to meeting cats’ basic needs and associated local site capacity and operations are summarised, with the results then considered relative to ADCH guidance where available.

### 3.1. Response Rates (n = 393 Total Responses)

Of the 961 identified organisations/sites, a total of 407 individual survey responses were collected, of which 14 duplicates were removed, leaving 393 unique responses. Response rates for the different survey sections varied from 73% (e.g., information about cat pen dimensions) to 100% (e.g., respondent demographics; cat socialisation and interactions). See Supplementary Materials (Document S2: Survey Results) for absolute numbers for responses used to calculate percentages for each question.

### 3.2. ADCH Membership (n = 393 Total Responses)

The majority of respondents (68.2%, *n* = 268) indicated that their organisation/charity was a member of the ADCH; 18.1% (*n* = 71) reported that their organisation/charity was not and 13.7% (n = 54) reported they did not know the membership status of their organisation/charity.

### 3.3. Locations of Sites Where Respondents Worked/Volunteered (n = 393 Total Responses)

**Figure 1:**
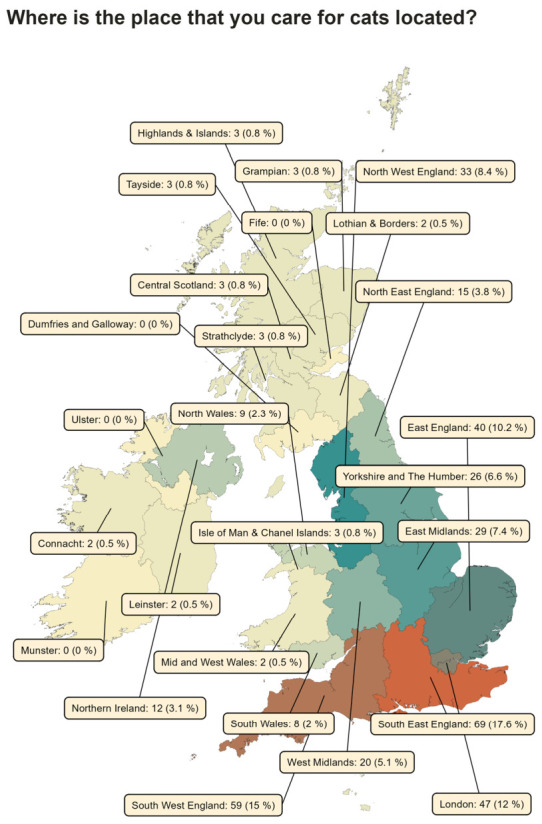
Regions within British Isles where respondents reported caring for cats, numbers for each region and their percentage of population as a whole.

Most of the shelters contacted were based in England (79.6%, *n* = 853), as were most of the individuals that responded to the survey (86%, *n* = 338), with the regions of the South East, South West, London and East England being particularly represented (see Figure 1).

The regional breakdowns in survey response rates compared to contacted organisations broadly mirrored each other, particularly in South East England (15.4% contacted vs. 17.6% responses) and East England (10.2% vs. 11.2%). This provides a crude estimate of relative geographic representation, with the caveats that multiple individuals from the same organisation could participate and that the analysis does not account for potential differences in organisation size or variations in the structure of the volunteer and foster networks across regions.

### 3.4. Local Site Environment (n = 393 Total Responses)

The most common environments where respondents reported providing care for cats were cattery/shelter/rehoming centres (56.5%, *n* = 222) and a room(s) within a private domestic residence (22.9%, *n* = 90). These were followed by a *collection* of pens near to or adjoining a private residence (12.5%, *n* = 49), a *single* pen near to or adjoining a private residence (6.9%, *n* = 27), and ‘other environments’ (1.3%, *n* = 5), e.g., a veterinary hospital or mixture of an indoor/outdoor environment.

### 3.5. Activities Undertaken at Local Site (n = 393 Total Responses)

Almost all respondents indicated that the site where they cared for cats offered temporary care and housing of cats and their subsequent homing (97.5%, *n* = 383). Other reported services that were less frequently undertaken included trap–neuter–return (42.5%, *n* = 167) and trap–neuter–relocate (35.1%, *n* = 138) of unowned cats, neutering of owned cats within the community (31.3%, *n* = 125), temporary care and housing of other species (26.5%, *n* = 104), and permanent care and housing of cats (13.0%, *n* = 51). The least frequent services were permanent care and housing of other species (5.9%, *n* = 23) and temporary care and housing of cats for commercial purposes (4.1%, *n* = 16).

### 3.6. Meeting Cats’ Basic Needs

#### 3.6.1. Group Versus Single Housing

##### Maximum Numbers of Cats Housed Together Within a Single Pen/Unit/Area (*n* = 364 Total Responses)

The largest percentage (60.7%, *n* = 221) of respondents reported the maximum number of cohabiting cats was 2, while 16.8% (*n* = 61) reported this was 3 cats and 10.6% (*n* = 39) reported between 4 and 10 cats.

##### Housing of Cats from the Same Previous Households/Environments Together (*n* = 364 Total Responses)

The largest percentage of respondents (58.8%, *n* = 214), reported that cats from the same previous households/environments were ‘usually’ housed together within a single pen/unit/area. A total of 42.6% (*n* = 152) of participants reported ‘always’ using the information about the relationships of cats in their previous living environment to determine whether to house them together.

Where cats appeared to be *actively struggling* in each other’s company (i.e., fighting, resource guarding or blocking), a large percentage of respondents (93.0%, *n* = 332) reported ‘always’ or ‘usually’ separating them. Comparatively fewer respondents (73.9%, *n* = 264) reported doing similar where cats did not appear to be *actively enjoying* each other’s company (e.g., lack of mutual playing, grooming, sleeping, and resting together).

##### Housing of Cats from Different Previous Households/Environments Together (*n* = 364 Total Responses)

The largest percentage of respondents (76.4%, *n* = 278) reported that cats from different previous households/environments were ‘never’ housed together within a single pen/unit/area. A total of 42.3% (*n* = 33) reported ‘always’ using information about the cats’ reported relationship with any other cats to determine whether they should be housed together with unfamiliar cats.

Where cats appeared to be *actively struggling* in each other’s company, a large percentage of respondents (91.1%, *n* = 71) reported ‘always’ or ‘usually’ separating them. Again, comparatively fewer respondents (79.5%, *n* = 62) reported doing similar where cats did not appear to be actively enjoying each other’s company.

#### 3.6.2. Pen Dimensions (*n* = 290 Total Responses)

Single cats: the median reported volume of pens for single cats was 11.34 m^3^ (IQR 7.56^3^–20.0 m^3^).

Pairs of cats: proportionately much less space per cat and only slightly more space overall was reported for pens housing pairs of cats, with a median volume of 12 m^3^ (IQR 8.0^3^–27.0 m^3^).

Cats housed in groups of three or more: the largest volumes were reported for cats housed in larger groups, with a median of 20.0 m^3^ (IQR 9.3–46.5 m^3^).

**Figure 2:**
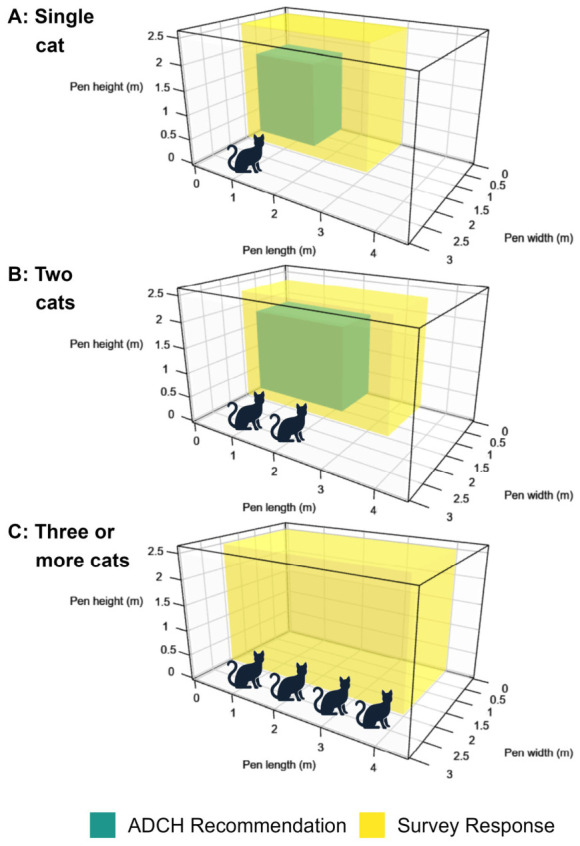
Yellow cubes show the median pen dimensions reported in the survey for single cats, pairs and groups of three or more, and green cubes represent relevant ADCH recommendations.

See Figure 2 for plots of pen dimensions. These are also plotted relevant to the ADCH’s guidance, with comparisons directly addressed in Table 1 of the Results Section.

#### 3.6.3. Resource Provisions

##### Types of Resources Provided and Their Frequency (*n* = 393 Total Responses)

**Figure 3:**
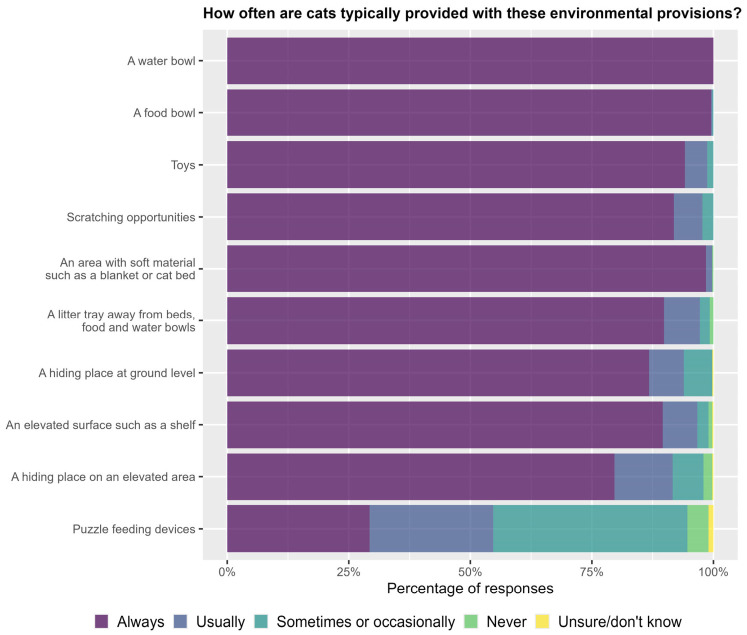
Frequency (and percentage) with which cats are provided with various environmental provisions in their pens/units.

Resources that were reportedly ‘always’ provided by all or the vast majority of respondents included a water bowl, food bowl, toys, scratching posts/opportunities and an area with soft material such as a blanket or cat bed. Resources where fewer respondents reported ‘always’ providing them included a litter tray located away from beds, food and water bowls, a hiding place at ground level and an elevated surface such as a shelf. The two least consistently provided items included a puzzle/enrichment feeding device and a hiding place on an elevated area. Across each of the aforementioned items, a small subset of respondents reported either ‘never’ or ‘sometimes’ providing these items (see Figure 3).

##### Number of Resources Provided for Single Cats (*n* = 393 Total Responses; Item-Level *n* = 372–393)

**Figure 4:**
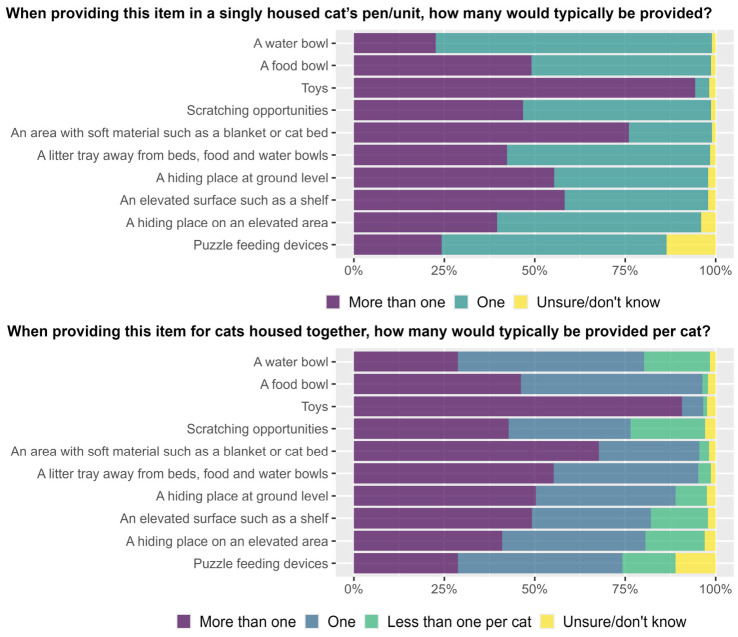
Rates (and percentage) at which different environmental provisions are included in single- and multi-cat pen/units.

Toys, followed by an area with soft material such as a blanket or cat bed, were the items respondents were most likely to report providing multiples of within a pen. For all other items, respondents were more likely to report providing only one, particularly for water bowls and puzzle/enrichment feeding devices (see Figure 4).

##### Number of Resources Provided for Group Housed Cats (*n* = 393 Total Responses; Item-Level *n* = 372–393)

As with singly housed cats, toys were the most commonly reported items being provided at a rate of more than one per cat, although comparatively fewer respondents reported similar for soft areas. The majority of respondents reported a one per cat or more than one per cat provision rate for litter trays away from beds, food, water bowls, and a hiding place at ground level. However, for all items, rates of less than one per cat were also reported (see Figure 4).

#### 3.6.4. Biosecurity and Cleaning Practices in Pens Occupied by Healthy Adult Cats

##### Cleaning of Food and Water Vessels (*n* = 361 Total Responses)

Almost all respondents indicated providing cats with fresh food (98.9%, *n* = 357) and water (97.5%, *n* = 352) in clean bowls every day.

##### Practices Occurring During a Typical Pen Clean (*n* = 361 Total Responses)

**Figure 5:**
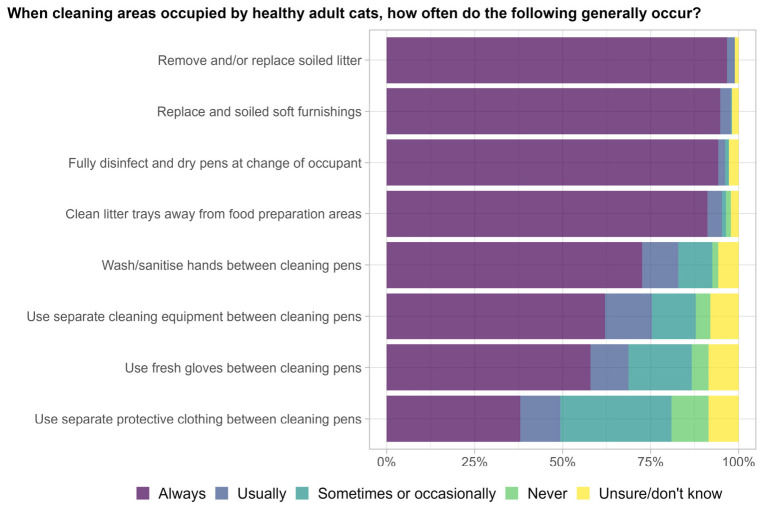
Frequency (and percentage) of biosecurity practices during cleaning of healthy adult cat pens.

A large percentage of participants reported ‘always’ removing any soiled litter from trays and replacing with fresh litter where needed; removing any soiled soft furnishings and replacing with clean items; fully cleaning, disinfecting and drying out a pen and any items within it at change in occupancy; and cleaning litter trays away from food preparation areas or outside of food preparation times (see Figure 5).

When moving between each pen during cleaning, the following practices were less consistently reported: washing or sanitising hands, using separate pen cleaning items/equipment, using separate gloves, and using separate protective clothing items (e.g., aprons, overshoes, overalls, or body suits) (see Figure 5).

#### 3.6.5. Managing Cat Psychological Distress During Cleaning and Husbandry Practices

##### Spot Versus Full Cleaning and Scent Continuity (*n* = 361 Total Responses)

**Figure 6:**
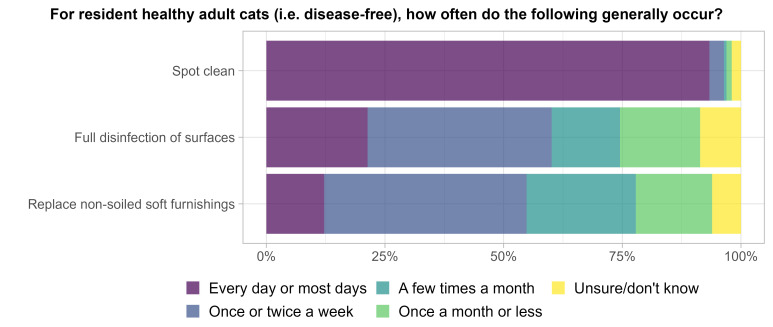
Regularity (and percentage) of spot cleaning, full cleaning, and replacement of non-soiled soft furnishings.

Concerning the regularity of *spot cleaning* of occupied pens (i.e., cleaning only dirty areas to reduce cat disturbance and support cat scent continuity), most respondents indicated that this occurred ‘every day or most days’. Smaller but substantial percentages of respondents also reported *fully cleaning* all/most vertical and horizontal surfaces within a cat’s pen with disinfectant ‘once or twice a week’ or ‘every day or most days’. Almost half of respondents reported removing and replacing clean, *non-soiled* soft furnishings (e.g., beds, blankets, and towels) within a cat’s pen was ‘once or twice a week’, with a smaller subset reporting undertaking this ‘every day or most days’ (see Figure 6).

##### Direct Handling of Cats During Pen Cleaning (*n* = 361 Total Responses)

**Figure 7:**
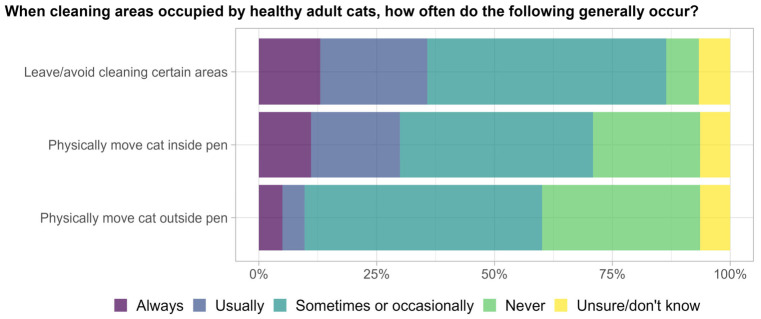
Frequency (and percentage) with which cats are handled and/or disturbed for routine cleaning.

The majority of respondents indicated ‘always’, ‘usually’ or ‘sometimes’ physically moving the cat to another area of their pen to facilitate cleaning. Over half of respondents also reported they would ‘sometimes’, ‘usually’ or ‘always’ physically place the cat into a carrier or another area outside of their pen. In contrast, fewer respondents reported ‘always’ or ‘usually’ leaving or avoiding cleaning certain areas so as not to disturb a cat (e.g., that is hiding/resting/sleeping) (see Figure 7).

#### 3.6.6. Preventative Health Care and Health Checks Prior to Homing (*n* = 368 Total Responses)

Almost all respondents indicated that cat parasite treatments (91.8%, *n* = 338) and vaccinations (89.9%, *n* = 331) were ‘always’ kept up to date at their shelters.

Almost all respondents (94.6%, *n* = 348) indicated that cats were ‘always’ seen by a registered veterinary surgeon (RCVS) or veterinary nurse (RVN) for a routine check/screening prior to homing. In terms of timing, 20.1% (*n* = 73) of respondents reported that cats were typically seen by a vet on their day of admission, 67.0% (n = 243) within their first week, and 3.1% (n = 11) within their first 10–21 days post-arrival. Amongst the respondents who did not indicate that cats were ‘always’ seen by a vet prior to homing, reasons mentioned included the following: (1) the cat was considered healthy or had been checked recently, (2) there were no veterinary appointments available or no time to see the vet, (3) the charity founders reportedly did not prioritise cat welfare sufficiently and (4) financial reasons.

#### 3.6.7. Cat Handling Techniques Used During Routine Health Checks (*n* = 347 Total Responses)

**Figure 8:**
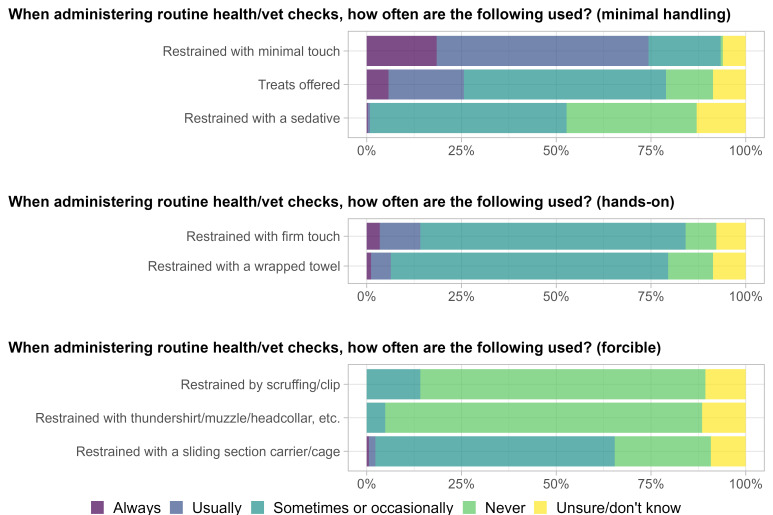
Frequency (and percentage) with which different restraint methods are used during routine health checks.

Most respondents indicated that cats were ‘usually’ or ‘always’ restrained using minimal touch or by lightly holding the cat’s body/head still with their arms/hands. Just over half of respondents reported that they ‘sometimes’ used treats to get the cat to remain still/to move into a desired position (see Figure 8).

Regarding more ‘hands-on’ approaches, most respondents reported ‘sometimes’ restraining cats by firmly holding the cat’s body/head still with the person’s arms/hands, as well as ‘sometimes’ restraining a cat by wrapping a towel around them (see Figure 8).

In terms of the more forcible restraint techniques, the majority of respondents reported ‘never’ using methods such as ‘scuffing’ or applying a clip to the back of a cat’s neck or using equipment such as Thundershirts, muzzles, or headcollars to restrict elements of a cat’s movement or behaviour. However, a small percentage of respondents reported that they ‘sometimes’ or ‘usually’ used these methods to restrain cats and the majority of respondents also reported they ‘sometimes’ or ‘usually’ used a sliding section or ‘crush’ cage to facilitate routine health checks (see Figure 8).

### 3.7. Local Site Capacity and Operations

#### 3.7.1. Caregiver Qualifications and Role-Based Training (*n* = 393 Total Responses)

Just over a third of respondents reported having a formal qualification in animal behaviour, welfare, health or animal training (34.4%, *n* = 135). Of those reporting qualifications, the most common was a BSc/level 6 qualification (30.2%, n = 32), followed by a level 3 qualification (17.0%, n = 18), with 11 respondents (10.4%) reporting a master’s/level 7 qualification.

The majority of respondents reported undertaking some type of work-based training in support of their current role, with ‘Understanding and meeting cats’ basic needs within the shelter environment’ (89.3%, *n* = 351), ‘Understanding and interpreting cat behaviour and body language’ (87.5%, *n* = 344) and ‘Interacting with and handling cats appropriately’ (85.8%, *n* = 337) being the most frequently selected options. Around half of respondents (ranging from 47.2% to 53.7%, depending on the topic) reported undertaking training within the last 12 months, with less (ranging from 16.5% to 21.4%) reporting this occurred within the last 1 to 2 years and fewer (ranging from 1.9 to 10.9%) within the past 2–5 years.

#### 3.7.2. Caregiver Fostering and Cat Ownership (*n* = 393 Total Responses)

The majority of respondents (61.1%, *n* = 240) reported providing foster care for cats within their private residences while working/volunteering for their current charity/organisation, of which most (67.9%, n = 163) reported that their premises were inspected (either in person or virtually) prior to fostering.

Most respondents (78.4%, *n* = 308) reported living with at least one cat or kitten of their own. Amongst the 306 respondents who lived with adult cats, 37.9% (*n* = 116) reported living with only one, 37.6% (*n* = 115) with 2 or 3, and 24.5% (*n* = 75) with between 4 and 21 cats. Two respondents lived only with kittens.

#### 3.7.3. Caregiver to Cat Ratios (*n* = 393 Total Responses)

Respondents reported that people caring for cats on a typical day at their site were responsible for a mean of 11.8 cats (sd = 10.1, median = 10), although values ranged from 1 to 67 cats per person, with over a third of respondents (38.1%, *n* = 132) reporting that people were usually responsible for caring for 11 or more cats daily.

#### 3.7.4. Number of Hours Worked/Volunteered per Week (*n* = 367 Total Responses)

The average reported volunteering hours were 19.1 h per week (sd = 16.9, median = 15); however, a small percentage of participants reported volunteering between 40 and 90 h (7.7%, *n* = 17). Five volunteers (2.3%) also reported volunteering for more than 90 h per week, but these were treated as erroneous data and not included in the calculations. Employees reported working on average 35.8 h per week (sd = 8.47, median = 35), although, again, a small percentage (13.0%, *n* = 21) reported working between 40 and 70 h per week. The average reported working hours were highest for individuals occupying ‘other’ roles (50.1 h per week (sd = 28.4, median = 40)), although individual values varied considerably from 1 to 90 per week.

#### 3.7.5. Waiting Lists and Intake Processes (*n* = 337 Total Responses)

The majority of respondents (88.1%, *n* = 297) reported that a waiting list was used to manage enquiries about cats to be admitted/brought into care, while 8.6% (*n* = 29) indicated that a waiting list was not used.

A similar percentage of respondents (81.0%, *n* = 273) reported using an assessment method or criteria to determine the *priority level* of the incoming cats, with comparatively fewer respondents (69.7%, *n* = 235) doing so to gauge cats’ *suitability*.

However, greater detail and more frequent mention of formal processes were mentioned regarding *suitability* assessment methods compared to *priority level* assessments. Reported methods used to assess *suitability* were mostly questionnaires/forms (mentioned by 21.9%, *n* = 40), usually completed by the owner/finder, and veterinary records/assessments (mentioned by 12.6%, *n* = 23). Half as many (10.1%, n = 21) respondents mentioned using questionnaires/forms, priority lists/guidelines, or tiering systems to assess intake *priority level*.

#### 3.7.6. Proportionate Pen Occupancy

##### Total Number of Separate Units/Pens Available (n = 291 Total Responses)

Respondents reported their sites having from 1 to 150 separate units/pens/rooms/areas to house cats, with a mean of 26.6 units per site (sd = 26.3, median of 18).

##### Number of Cats on Waitlists as a Proportion of Separate Units/Pens Available (*n* = 219 Total Responses)

The mean reported number of cats per *waiting list* was 58.9 cats (sd = 75.3, median = 29, range = 0 to 450). The median proportion of cats on the waiting list as a proportion of the number of separate units/pens available was 2.22 (IQR = 3.43, range = 0–142.5).

##### Numbers of Cats Currently in Care as a Proportion of Separate Units/Pens Available (*n* = 322 Total Responses)

The mean reported number of cats *currently in care* was 35.6 (sd = 33.9, median = 26.5, range = 0 to 200). The median number of cats currently in care as a proportion of the total number of separate units/pens available was 1.34 (IQR = 1.36, range = 0–27.5).

##### *Maximum* Number of Cats Housed as a Proportion of Separate Units/Pens Available (*n* = 204 Total Responses)

The mean number of *maximum* cats that were housed at one time over the preceding 12 months was 37.9 (sd = 38.2, median = 30, range = 1 to 190). At that time, the median number of cats in care as a proportion of the total number of separate units/pens available was 1.78 (IQR = 2.26, range = 0.05–23.3).

#### 3.7.7. Emergency Intake (*n* = 337 Total Responses)

Comparatively low numbers (15.7%, *n* = 53) of respondents reported there were ‘always’ units/pens/areas at their site kept empty and reserved for emergency admission/intake, with similar numbers (18.1% (*n* = 61) reporting these were ‘never’ available. More respondents reported units were either ‘sometimes’ (36.8%, *n* = 124) or ‘usually’ available (21.1%, *n* = 71). The remainder (8.3%, *n* = 28) reported they were ‘unsure/didn’t know’.

#### 3.7.8. Isolation Facilities (*n* = 368 Total Responses)

The majority of respondents (89.1%, *n* = 328) reported that there were options for cats to be placed in isolation facilities (i.e., enabling individuals with suspected or confirmed contagious disease to be kept in a self-contained pen/unit/room/area away from healthy cat populations). A smaller percentage indicated that there were no isolation facilities (6.8%, *n* = 25) or that they were ‘unsure/didn’t know’ (4.1%, *n* = 15).

#### 3.7.9. Cat Length of Stay

##### Cats Homed as Pets/Human Companions over the Past 12 Months (*n* = 333 Total Responses)

**Figure 9:**
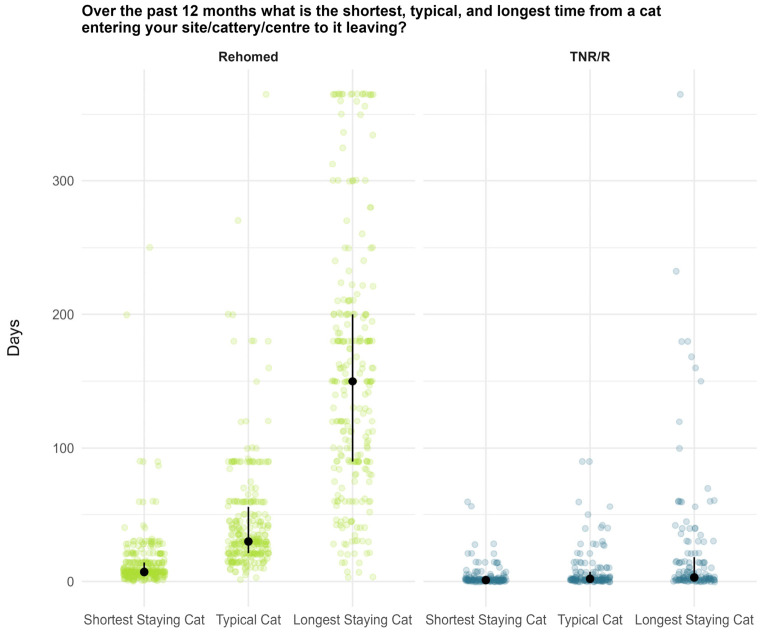
Distribution of reported minimum, typical, and maximum lengths of stay for rehomed and managed via TNR/R. Coloured points show individual responses; black points and vertical bars indicate median values with IQR.

Median reported stays for pet cats were 30 days for a typical cat (IQR = 37.2, range = 1–365), 7 days (IQR = 9, range = 0–25) for the shortest staying cat and 150 days (IQR = 120, range = 3–365) for the longest staying cat. Large variations in reported lengths of stay across each category were evident, including long lengths of stay reported by a number of individuals (see Figure 9).

##### Cats Trapped, Neutered and Returned/Relocated (TNR/R) over the Past 12 Months (*n* = 206 Total Responses)

Median reported stays for TNR/R (i.e., most likely ‘feral’) cats were 2 days (IQR = 6, range = 0 to 90) for a typical cat, 1 day (IQR = 1, range = 0–60) for the shortest staying cat and 3 days (IQR = 17, range = 0–365) for the longest staying cat. Large variations in reported lengths of stay across each category were, again, evident, including long lengths of stay reported by a number of individuals (see Figure 9).

### 3.8. Sector Alignment with ADCH Minimum Standards

Relevant minimum ADCH standards were available for comparison to survey results for most areas, with the exception of cat handling, staff working hours, and proportionate pen occupancy. Majority alignment was considered present where more than 50% of respondents reported approaches consistent with relevant guidance. The following table presents a summary of evidence for each survey subsection, with the results suggesting relevant minimum standards were either met or partially met by the majority (i.e., >50%) of respondents. However, for certain areas, particularly cat-to-caregiver ratios, minimum standards were available but could not be directly compared to the results. This was due to the limited parameters included within the guidelines to facilitate direct comparisons.

**Table 1:** Evidence summary to highlight where relevant ADCH standards were either met or partially met by the majority (i.e., >50%) of respondents. See Supplementary Document S3: ADCH Minimum Requirements for an expanded version of the current table, which also includes full details of each relevant minimum standard.

**3.6 Meeting Cats’ Basic Needs**	
**Survey Subsection:**	**Evidence of Relevant ADCH Minimum Standard Met**
3.6.1 Group versus single housing	Evidence minimum standards partially met by majority; more than 50% of participants reported consistently avoiding housing unfamiliar cats together and consistently separating cats not getting on well together. However, less than 50% of respondents reported consistently using previous social history of cats to inform social housing decision-making
3.6.2 Pen dimensions	Evidence minimum standards met by majority for single- and pair-housed cats; calculated median volumes reported in the survey were greater than those based on ADCH recommended dimensions. ADCH guidelines do not provide any recommended values for cat groups larger than four; therefore, our results regarding volumes for cats housed in groups of 3 or more cannot be directly compared to ADCH guidance.
3.6.3 Resource provisions	Evidence minimum standards partially met by majority; more than 50% of respondents consistently provided basic resources, with the exception of feeding enrichment. Over 50% of respondents reported providing one or more than one of each resource per cat where multi-housed.
3.6.4 Biosecurity and cleaning practices in pens occupied by healthy adult cats	Evidence minimum standards met by majority; over 50% of respondents reported providing clean food and water bowls daily, cleaning pens daily or most days, consistently removing soiled items and litter during cleaning, fully cleaning, disinfecting and drying and pens at change in occupancy and cleaning trays away from food preparation areas.
3.6.5 Managing psychological distress during cleaning and husbandry practices	Evidence minimum standards partially met by majority; over 50% of respondents reported regularly spot cleaning; however, over 50% also reported regular replacement of non-soiled soft furnishings and regular/semi-regular full disinfection of pens, as well as direct handling and disturbance of cat during cleaning.
3.6.6 Preventative health care and health checks prior to homing	Evidence minimum standards met by majority; over 50% of respondents reported parasite treatments and vaccinations kept up to date, and over 50% of respondents reported cats consistently seen by a vet prior to homing and either on the first day or within first week of arrival.
3.6.7 Cat handling during routine health checks	No standards available for comparison.
**3.7 Local site capacity and operations**	
3.7.1 Caregiver qualifications and role-based training:	Evidence minimum requirements met by majority; less than 50% of participants reported holding a relevant formal qualification; however, over 50% of participants reported undertaking work-based training on animal-welfare related topics in support of their current role and within the past 12 months. The guidelines do not specify how often training should be undertaken or the specific types of training or qualifications that are recommended.
3.7.2 Caregiver fostering and cat ownership	Evidence minimum standards partially met by majority; over 50% of participants reported premises inspected prior to fostering; however, parameters for determining overcrowding or hoarding risk not specified and, therefore, cannot be compared against reported cat ownership rates, etc.
3.7.3 Caregiver-to-cat ratios	Standards do not specify values, therefore, comparison not possible.
3.7.4 Number of hours worked/volunteered per week	No standards available for comparison.
3.7.5 Waiting lists and intake processes	No standards available for comparison.
3.7.6 Proportionate pen occupancy	No standards available for comparison.
3.7.7 Emergency intake	No standards available for comparison.
3.7.8 Isolation facilities	Evidence minimum standards met by majority; over 50% of participants reported there were options for cats to be placed in isolation facilities.
3.7.9 Cat length of stay for TNR/R (i.e., ‘feral’) cats	Evidence minimum standards met by majority; median reported 2 day stay for an average ‘feral’ cat (i.e., those managed under TNR/R pathways).

## 4. Discussion

### 4.1. General Summary

A survey of the British Isles shelter sector was undertaken in 2023 in order to quantify current approaches to cat housing, husbandry, and general cat care practices across the British Isles shelter sector. The current manuscript reports on practices relevant to meeting shelter cats’ basic needs and local site capacity and operations, considering these in relation to industry standards (i.e., ADCH guidance [34]) where possible. As a whole, the survey results suggested evidence of positive approaches across the sector in relation to meeting cats’ basic needs, including majority (i.e., over 50%) alignment to elements of the ADCH’s minimum standards where comparable. However, substantial variation in reported cat care practices were also captured, including areas where cats’ needs could be met more consistently and where local capacity may exceed demands, potentially compromising cat and human wellbeing as a consequence. Additionally, current ADCH standards provide mostly top-level guidance rather than more prescriptive detail, limiting direct benchmarking of survey results. These points are expanded in the following sections, followed by suggestions to support both site- and sector-level improvements.

### 4.2. Meeting Cats’ Needs in the Shelter Environment

In general alignment with relevant ADCH guidance, almost everyone reported consistently keeping cats up to date with their parasite treatments and vaccinations, and more than 50% reported consistently providing most essential resources within pens (e.g., food and water bowls, scratching posts, litter trays and beds) and in multiple quantities where cats were housed together. The majority also reported applying appropriate biosecurity measures, such as removing or replacing soiled items in pens, good litter tray hygiene, and full pen cleans at change in occupancy.

However, there was also considerable variability in responses, including small to substantial percentages of responses that reflected practices likely to limit the meeting of cats’ psychological needs in particular. For example, in terms of resource provisions, despite their importance in managing stress and anxiety and promoting positive wellbeing [10,57,58,59,60,61], a considerable percentage of respondents reported inconsistently providing cats with a hiding space, elevated surfaces, a litter tray placed away from other resources and puzzle/enrichment feeding devices. For certain resources, provision rates of less than one per cat in multi-cat pens were also reported. Additionally, in terms of cat handling and husbandry practices, percentages of respondents reported applying potentially aversive handling methods and equipment, as well as cleaning practices that may disrupt a cat’s sense of security [4,5,62,63].

In relation to pen sizes, ADCH guidance was sufficiently prescriptive to enable direct comparisons with survey data, with the median calculated survey pen volumes for cats housed singly and in pairs substantially larger than those calculated based on ADCH guidance. This suggest that, on average, the sector is exceeding minimum standards for total space provision for individual and pair-housed cats. However, paired housed cats within the survey had proportionately much less space than those housed singly. Additionally, in the absence of a robust scientific evidence base for optimal pen dimensions and without considering pen volumes in the broader context of holistically meeting cats social and environmental needs, it is difficult to determine whether shelter compliance with recommended sizes translate to direct welfare benefits for cats on an individual level.

For example, cats’ access to three-dimensional space, such as the provision of elevated areas of different heights and the ability to move easily between these, constitute core environmental needs [45,64,65]. Therefore, without sufficient internal structures provided within pens (such as staggered shelving, chairs, tables, ladders and climbing trees), much of the vertical volume within a pen may be of redundant value, essentially reducing the amount of total pen space the cat has physical access to and can directly benefit from. Cats also demonstrate strong preferences for relatively enclosed spaces where they have the ability to remain obscured from view, and the provision of hiding opportunities is considered another core environmental need [9,45,58]. Therefore, if hiding opportunities and enclosed areas are not provided at a suitable rate proportionate to pen volume (i.e., the larger the area, the more hiding options need to be provided regardless of absolute cat numbers), greater total pen volume is unlikely to directly equate to better cat wellbeing. Indeed, a large open-space pen with limited options for elevation and concealment may be particularly stressful for cats, impeding acclimatisation or even inducing chronic stress [61]. On the other hand, if pens are appropriately furnished, larger pen sizes are likely to provide cats with a greater sense of choice and control over their environment [44,66]. Similarly, where cats are housed with conspecifics, the provision, and careful placement, of multiple resources to reduce competition and conflict is important [45], and this is potentially easier to achieve with a larger pen size, if suitably furnished [64]. However, as survey responses indicated inconsistencies in the provision, quantity and placement of basic pen resources for both singly and group housed cats, this does introduce uncertainty over the direct benefits to cats regarding pen sizes and compliance to ADCH standards. More detailed guidance on appropriate furnishing, resource provisioning and use of three-dimensional space within pens relative to their size and dimensions would be useful.

In relation to decisions around housing cats together, survey responses appeared to reflect partial alignment with the relevant ADCH guidance, which states the following: “*Single housing is the best choice for cats unless they have been living together harmoniously in a home previously*” and that “*Putting together unrelated or incompatible cats can put pet cats under pressure and cause stress*.” Most respondents reported ‘never’ mixing cats from different previous living environments and consistently separating cats actively struggling in each other’s company. However, when it came to initial decisions about whether to house cats together, less than half of respondents reported using information about the cats’ previous relationships with conspecifics in their decision-making. Additionally, in terms of decisions over whether to separate cohabiting cats, signs of overt conflict appeared to be prioritised over more subtle presentations of cats not enjoying each other’s company, with cats from the same previous environments also being less likely to be separated, compared to unfamiliar cats housed together. Such populations may, therefore, be at risk of compromised wellbeing, particularly if they are also being exposed to inadequate pen provisions [57,67,68] and/or are housed in larger groups (as reported by a percentage of respondents). More detailed practical guidance around collecting and using information about a cat’s previous living environments to support decision-making, optimal resource provisioning and behavioural monitoring could be useful to shelters.

In relation to managing psychological distress during cleaning and husbandry practices, a large percentage of respondents reported regularly undertaking cleaning-related activities which would likely reduce cats’ perceived sense of safety and security, and could seemingly deviate from relevant ADCH guidance. For example, guidance states “*Cleaning and husbandry practices need to take into consideration the psychological distress that may be caused by the daily removal of familiar smells and bedding through the use of large amounts of water and disinfectant…Housing or bedding that is too rigorously cleaned may remove valuable scent marking. The use of a disinfectant-led spot cleaning approach satisfies the demands of both welfare and hygiene once the animal has gone through the quarantine period*”. While most people reported regularly ‘spot cleaning’ pens, they also reported regularly fully cleaning most/all surfaces in the cat’s pen, changing clean bedding items, and physically moving cats from where they may be hiding/resting/sleeping and/or placing them into cat carriers to facilitate cleaning. Given that cats use their scent to enhance their sense of familiarity and security [63] and that they are sensitive to unpredictable handling and husbandry routines that limit autonomy [4,5,62], such approaches to cleaning may lead to increased risk of stress and disease [4,46,69]. More detailed practical guidance on effective cat-friendly approaches to cleaning and husbandry could therefore be useful to shelters.

### 4.3. Local Site Capacity and Operations

Reported rates of work-based training were high with over half of respondents indicating they had received training within the 12 months prior to taking the survey. The results suggest surface alignment with ADCH guidelines, highlighting efforts within the sector to ensure cat caregivers have up to date levels of knowledge and expertise to undertake their roles. However, ADCH guidelines do not specify how often training should be undertaken or the specific types of training or qualifications required, and there are currently no relevant formally accredited training courses or protected role titles for benchmarking. It is, therefore, difficult in practice to determine if and what types of knowledge gaps and associated training needs may or may not be present within the sector.

In relation to cat fostering, ADCH guidance recommends foster premises are visited and checked prior to fostering. While over 50% of respondents that fostered cats reported their premises were inspected prior to fostering, this still left a large percentage where inspections did not take place. Additionally, recommendations are that “*An assessment shall be made, and steps in place to avoid the risk of over crowding and hoarding both owned and foster cats*”. However, definitions of ‘overcrowding’ or ‘hoarding’ are not provided within the standards and therefore may be difficult to identify and regulate in practice, particularly given that there is no agreed value within the scientific literature regarding the numbers of animals that would constitute a case of either. For example, in a paper documenting the characteristics of hoarders [70], the mean number of hoarded animals was 50, however reported numbers of household cats varied from one to 75. While in the current survey, almost a quarter of respondents reported living with between 4 and 21 cats of their own, more qualitative information about the wellbeing and living conditions of both the privately owned and foster cats would be required before determining whether individuals were being subjected to over-crowding/hoarding or not, e.g., [71]. This is where practises such as undertaking in-person or virtual premises inspections (by a suitably competent individual as per ADCH standards) to identify such cases would be extremely useful. However, this may be also problematic to implement, given the lack of detailed guidance within the ADCH standards (and elsewhere) concerning what constitutes a suitable skill/competency level to be able to undertake such assessments.

In relation to shelter cat to staff ratios, ADCH standards do not include numeric guidance on recommended maximum cat to staff numbers but instead state “*consideration must be foremost given to ensuring that there is enough capacity between all people to be able to provide the five welfare needs for every individual animal onsite.*” In the current survey, some respondents reported individually caring for up to 67 cats, arguably a very high number to be able to sufficiently meet the individual daily welfare needs for each individual. However, is impossible to determine whether this amount would contravene current guidance, given the standards do not provide specific detail on how to determine sufficient caregiver capacity or whether the five welfare needs for each individual are being met. The provision of further detailed guidance in these areas would, therefore, be beneficial to shelters.

An area not covered within ADCH guidance but where responses raised caregiver capacity concerns included reported working practices. A percentage of respondents indicated working between 40 and 70 h per week or volunteering between 40 and 90 h per week. Given the prevalence of burnout and compassion fatigue within the animal care sector [42,43], further research is recommended in order to understand the practical contexts, pressures and motivations that may be driving people to work such long hours and subsequent guidance and interventions developed.

In relation to the housing of ‘feral’ cats, ADCH guidance states; “*Feral cats must not be kept in confinement any longer than 48 h, unless immediate veterinary intervention is needed.*” The median length of stay over the past 12 months for TNR/R cats (i.e., those likely deemed by respondents to be ‘feral’) was 2 days, thus aligning well with guidance at the population level. However, reported values were highly variable between individual respondents and, in some cases, reached the maximum response option of 365 days, thus greatly exceeding the 48 h stipulated by ADCH. Given the impact of human proximity and long-term confinement on the wellbeing of cats unsocialised to humans [25], these long lengths of stay for such cats are concerning. Further research is recommended in order to understand why some cats experience very long stays in shelters before being managed via TNR/R, and what interventions may help reduce their time in care.

On average, there were slightly over two cats on the waiting list for every separate unit available to house cats at a site. However, values were very variable, with some respondents reporting up to 142 cats for every separate unit, suggesting disparate demand at local levels and very high demand versus available resource in certain instances. There were also on average 1.34 cats reported as currently being in care for every separate unit, increasing to 1.78 per unit at their busiest point. Individual values were, again, very variable from 0 to 27.5 cats per separate unit. These results indicate that, in practice, many sites are likely to struggle to provide individual housing to the majority of cats in their care as standard, highlighting important risks of stress, overcrowding and disease [8,11,24] and, potentially, deviation from various ADCH standards. These welfare risks may be compounded where waiting lists or formal processes to manage intake are not used and where there is limited access to isolation facilities, empty pens for emergency intake and veterinary care, as reported by some respondents.

Combinations of the aforementioned factors would likely create substantial capacity for care challenges, where cat occupancy essentially exceeds the local resources required to manage populations effectively and provide good cat welfare standards [20]. Given that operating in this way can also be associated with poorer homing efficiency e.g., [3,72], it is possible that these types of factors may be contributing to the high lengths of stay for both companion cats and those deemed to be ‘feral’ that were reported by some respondents. Impacted sites could potentially benefit from the application of targeted shelter population management models to optimise their capacity for care, subsequently enhancing cat welfare, reducing lengths of stay and leading to greater numbers of cats being helped overall [19,20]. It is also likely that sufficiently addressing capacity for care issues may protect against staff burnout and compassion fatigue, reducing the pressure placed on individuals and their perceived need to work such long hours [19]. Greater sector-wide collaboration may also help to mitigate against wider barriers to shelter cat flow and drivers of intake, helping to reduce demands placed on individual sites.

### 4.4. Study Limitations

We were successful in capturing substantial diversity both in terms of respondent demographics and the nature and structure of organisations and sites reported on (see Supplementary Materials Document S2: Survey Results for full details). However, given the anonymised way data were collected, multiple people within a single organisation or at a single site were able to complete the survey. The results should, therefore, be considered to provide an initial descriptive overview into current practices and their variation at the individual site level, rather than a true proportionate representation of the sector as a whole.

Direct comparison of responses across different shelter sizes, structures, geographic locations and between ADCH members and non-members was not undertaken. This was in order to avoid multiple subgroup analyses per question, keeping data analysis and reporting to an appropriate length and preventing unbalanced/small sample sizes that did not allow for robust comparisons. Future work could therefore explore potential associations between these demographic features and approaches to cat care. Comparisons between sites in regions where current licencing is (Scotland) and is not (England and Wales) in force would be beneficial, as would pre- and post-licencing comparisons within the same area if greater licencing of the British Isles sector occurs in the future.

The survey aimed to comprehensively explore variations in cat care across the sector, prioritising areas considered most relevant to cats’ individual welfare experiences. In doing so, however, more detailed aspects of housing and husbandry (e.g., qualitive details regarding pen furnishings, the predictability of daily routines, noise levels in the cattery, specific types of enrichment offered) were not captured. This was considered an acceptable compromise, given the need to cover a broad range of areas (including approaches to behaviour and welfare assessments, to be covered in future manuscripts), as well as avoid respondent survey fatigue. However, it is important to highlight that the various parameters reported on in this survey were all relatively top-level and based on respondents’ perspectives, thus potentially lacking the granularity and objectivity required for them to be used as good proxies for cat care and welfare standards. For example, while the majority of people reported consistently providing cats with hiding options, it was not possible to determine whether these ‘hiding’ resources represented suitable opportunities for hiding *from the cats’ perspectives* (i.e., offering sufficient concealment, placed in a suitable location) or merely from the humans’.

For practical reasons, survey methodology relied on participant self-selection and self-reporting. Survey anonymisation and careful wording of questions/answer options was undertaken to avoid unduly influencing participants’ responses. However, there is the potential for responses to be biased towards individuals/organisations with a greater interest in cat welfare and for responses to be skewed towards more welfare-friendly answers. For a more detailed understanding of the current standards of cat welfare across the sector, more objective, environment and cat-based indicators are required. Suitably practical, well-validated tools to achieve this are not yet available, however, given the inherent complexities associated with welfare assessment, e.g., [73,74].

## 5. Conclusions and Future Recommendations

Overall, survey results suggest evidence of positive approaches to basic cat care across the British Isles shelter sector, including degrees of alignment to ADCH minimum standards where comparable. However, substantial variation in certain practices were also identified, highlighting potential deviations from best practice. Collectively, these findings can be used to support the development of targeted training, resource allocation and further guidance to improve the consistency in meeting the needs of shelter cats at local levels.

### 5.1. Suggested Resources to Support Site-Level Approaches to Cat Care

The development of detailed practical guidance and training in the following areas is recommended to ensure consistent positive site-level cat care:

- Multi-cat housing, monitoring and associated decision-making;
- Pen furnishing and resource provisioning for single- and multi-housed cats, optimised for relative pen size and dimensions;
- Appropriate biosecurity approaches which reduce physical disturbance, avoid direct handling of cats and promote scent continuity;
- Approaches to low-stress handling methods during cat health checks;
- Guidelines for establishing site-level capacity based on resources and ability to meet cats’ welfare needs including appropriate cat intake, assessment and population management processes, cat-to-staff ratios, staff working hours, cat lengths of stay and access to isolation and emergency intake facilities.

### 5.2. Implications for Future Legislation and Sector Guidance

This research is particularly timely considering the UK government position regarding the need for additional shelter sector guidance and regulation, and the current animal shelter licencing bill under consideration [75,76]. It is important to highlight that shelter compliance with top-level regulations, including any future licencing requirements, may not necessarily directly translate to better wellbeing experiences from the cats’ perspective, if a holistic approach to housing and husbandry grounded in good understanding of cat behaviour and welfare is not applied in practice.

To this effect, formal professionalisation of the sector and development of accredited courses targeted to specific roles and their essential knowledge requirements may be beneficial. The development of comprehensive, detailed guidance on best-practice approaches to cat shelter housing and husbandry, in addition to the application of suitable cat and environment evaluation and impact measures could also be helpful. Such approaches could help to ensure that ‘best-practice’ and ‘capacity for care’ principles are applied comprehensively within shelters, including important areas not currently covered by ADCH standards and/or those that may be difficult to sufficiently embed within standard licencing frameworks. To understand how the sector may be best supported to improve shelter cat care and to create a more standardised landscape, it is also important to fully engage with its stakeholders. Exploring current barriers at both local and national levels and incorporating the needs and perspectives of stakeholders into any planned interventions is vital.

## Data Availability

The data presented in this study are openly available in the Open Science Framework at https://osf.io/x4gmf/?view_only=92148f18672f49c29281bd5fc786322d (accessed on 21 May 2026).

## Supplementary Materials

The following supporting information can be downloaded at: https://www.mdpi.com/article/10.3390/vetsci13060587/s1, Document S1: Survey Questions; Document S2: Survey Results; Document S3: ADCH Minimum Requirements.

## Abbreviations

The following abbreviations are used in this manuscript:

UK	United Kingdom
ADCH	Association of Dogs and Cats Home
TNR/R	Trap–Neuter–Return/Relocate
NTU	Nottingham Trent University
ARES	Animal Rural and Environmental Sciences
GDPR	General Data Protection Regulation
