# Supplementary material for: Part 1: A Sector-Wide Survey of UK/British Isles Shelter Organisations Caring for Cats: Caregiver-Reported Approaches to Housing, Husbandry and General Care Provision
Source: Vet Sci. 2026 Jun 16;13(6):587. doi: 10.3390/vetsci13060587 (PMC13308460; doi:10.3390/vetsci13060587)
Supplement: Supplementary file 1 [file vetsci-13-00587-s001.zip › Document S1 - Survey Questions.pdf]

Document S1: Survey Questions

iStartx1

Please help us to better understand cats across shelters and develop future cat wellbeing tools!

You are being invited to participate in a research study developed by cat welfare researchers at Cats Protection. Participation will involve completing an online survey and answering questions about shelter cat housing and husbandry. This important information will be used to help the research team to develop future cat wellbeing assessment tools to support both cats and their carers across the shelter sector.

The survey will take around 15-45 minutes to complete depending on how many sections you answer. You will be able to save your progress through the survey so that you can complete it over multiple occasions if preferred. You can also skip any sections you might not immediately know the answers to or return to answer these questions at a later date when you have the necessary information.

As a thank you we are offering the first 700 eligible* participants to complete the survey a free £20 Pets at Home voucher. All participants eligible* will also have the option to enter into a prize draw for the chance to win:

An online CatCare Cat Friendly Homing course worth £695
An Association of Dogs and Cats Homes (ADCH) 2024 conference place worth around £152/£375 for members/non-members
One of 10 International Cat Care health or behaviour courses, each worth £99

Distribution of this survey is kindly supported by the Association of Dogs and Cats Homes (ADCH). A big thank you to ADCH, Pets at Home and International Cat Care for very kindly contributing towards the survey prizes*

*To be eligible, participants must be aged 18 years or over and be involved in the regular care provision of cats for a charity or organisation located within the British Isles, either as a volunteer or staff member

** These organisations have not been involved in the design or contents of the survey and will not have access to any of the raw survey data

This survey will close on 1st October. If you would like to participate, please ensure you have completed and submitted it by this date.

INSERT LOGOS OF ASSOCIATIONS SUPPORTING THIS SURVEY ON THIS PAGE

IStartx2

General Information for participants:

What is the study about? **SHOW HEADERS WITH STIM NEXT TO IT, CLICK TO ENLARGE (STIM 6)**

Who is undertaking the study? **SHOW HEADERS WITH STIM NEXT TO IT, CLICK TO ENLARGE (STIM 7)**

Who can take part in this survey? **SHOW HEADERS WITH STIM NEXT TO IT, CLICK TO ENLARGE (STIM 8)**

What kinds of questions will I be asked? **SHOW HEADERS WITH STIM NEXT TO IT, CLICK TO ENLARGE (STIM 9)**

Can I save my progress and complete the survey over multiple occasions? **SHOW HEADERS WITH STIM NEXT TO IT, CLICK TO ENLARGE (STIM 10)**

How will the data from the survey be used and will I be able to see it? **SHOW HEADERS WITH STIM NEXT TO IT, CLICK TO ENLARGE (STIM 1)**

Research T team

How can I opt out of the survey? **SHOW STIM WHEN CLICKED TO ENLARGE (STIM 2)**

Alternatively you can contact sheltersurvey@cats.org.uk at any point in order for your contact details to be removed from our database.

Information about study ethical approval and General Data Protection Regulations (GDPR): **SHOW STIM WHEN CLICKED TO ENLARGE (STIM 3)**

How Basis protect your privacy **SHOW STIM WHEN CLICKED TO ENLARGE (STIM 4)**

If I have any questions or concerns about the survey, who can I contact?

Please direct any questions or concerns to the Feline Welfare Research team sheltersurvey@cats.org.uk

QEligibilty

Eligibility and informed consent:

To be eligible to complete this survey, you must meet all the following criteria:

- Currently work or volunteer with an animal rehoming/rescue/shelter/sanctuary organisation within the British Isles, either full or part time. This includes anyone that might run their own rehoming/rescue/shelter/sanctuary organisation.
- Be directly involved in the regular provision of care to cats (other than those you own) that are being housed by or on behalf of the above organisation. Care provided may include routine housing and husbandry and/or behaviour and welfare assessments and cat welfare support
- Be aged 18 years or over

Please tick all that apply: **FORCE RESPONDENTS TO ANSWER ALL 3 BEFORE PROCEEDING**

- ☐ I confirm that I meet all of the above criteria
- ☐ I understand that my participation in this survey is completely voluntary and that my data will be fully anonymised prior to its analysis
- ☐ I give consent for the data I provide in this survey to be used for cat wellbeing related research purposes including research publications and academic talks
- ☐ I do not wish to participate in this survey **EXCLUSIVE, SCREEN OUT**

QEmailPII SHOW ON SAME PAGE AS QEligibility

Please provide us with an email address. This is required in order to complete the survey. Once entered, a link to your saved survey will be sent to you, enabling your progress through the survey to be automatically saved. Once you submit your survey, your data will no longer be linked to any email address you have provided here.

**Email address:**
**I do not wish to provide an email address (if ticked, you will not be able to progress with the survey)**

QEmailAgain

ASK THOSE WHO DO NOT WISH TO PROVIDE AN EMAIL ADDRESS, SC	
**Are you sure you do not wish to provide an email? You will not be able to complete the survey if you do not provide one.**	**Yes** SCREEN OUT
	**No** DIRECT BACK TO EMAIL ADDRESS QUESTION

iSurveyInstructions

Survey instructions:

- Please answer all questions in relation to the cats that you care for as part of the charity/organisation you work or volunteer with, rather than in relation to your own cats.
- If you care for cats for more than one charity/organisation, please choose the organisation that you spend the most time working or volunteering with.
- If you provide care for cats across multiple different physical environments for the same organisation, please answer in relation to the specific location (e.g. foster room/pen/site/cattery/centre) where you spend most time caring for cats.

What if I don't know the answer to a question in the survey?

SHOW STIM WHEN CLICKED TO ENLARGE (STIM 5)

Section 1: Details about your charity/organisation and your role

INFO: SHOW ALL

This section asks some questions about your role and the organisation you work or volunteer with. **You will not be asked to provide any identifying details about yourself or your organisation**.

This section took participants an average of 3 minutes to complete.

Question 1, SC, ASK ALL	Answer options ROWS	Tick all that apply
Is your *primary* role within your charity/organisation:	A paid position	
	A voluntary position	
	Other (please provide some brief details)	OE

Question 2, ASK ALL OE<br>*Please provide an answer to the nearest whole hour.*	Answer options RANGE 1-168 HOURS
How many hours a week on average do you work or volunteer with your charity/organisation?	Open numeric box
	Unsure/Don't know

Question 3, ASK ALL, MC	Answer options ROWS	Tick all that apply
What cat care activities do you regularly undertake as part of your role?	Feeding cats	
	Cleaning cat pens and associated equipment/areas	
	Socially interacting with cats	
	Undertaking behaviour and/or welfare assessments of cats	
	Implementing cat training or other behavioural modification	
	Providing support to other care staff/volunteers in the form of cat behaviour and/or welfare advice	
	Managing or supervising individuals who provide regular care for cats	
	Training individuals who provide regular care for cats	
	Involved in cat intake/admission	
	Involved in cat adoption/rehoming	
	Kitten hand rearing	
	Other (please provide some brief details)	OE, FIXED

Question 4, ASK ALL, SC	Answer options	Tick one	Question 5, ASK ALL WHO CODE Q4=YES	Tick one, SC
Do you currently provide or have you previously provided foster care for cats within your private residence while working or volunteering for your current charity/organisation?	Yes		Were your premises inspected (either in person or virtually) and assessed before you were able to foster cats?	Yes
				No
				Unsure/don't know/can't remember
	No			

Question 6, ASK ALL, MC	Answer options ROWS	Tick all that apply	
Have you undertaken any of the following types of training in support of your current role?	Understanding and meeting cat's basic needs within the shelter environment		
	Recognising good and also poor welfare in cats in the shelter environment		
	Understanding and interpreting cat's behaviour and body language		
	Interacting with and handling cats appropriately		
	Disease prevention, recognition and outbreak control		
	Pen and associated equipment cleaning		
	Any other cat care training (please provide some brief details)		OE
	Unsure/ can't remember	EXCLUSIVE	
	No training undertaken	EXCLUSIVE	

Question 7, GRID, SC PER ROW<br>[q7 to appear if ticked anything other than 'no training' or 'unsure/don't know] for Q6:	Tick one, COLUMNS	
When did you last receive any of this type of training?<br><br>INSERT SELECTED STATEMENTS AT Q6 FOR ROWS	Within the last 12 months	
	1-2 years ago	
	2-3 years ago	
	3-4 years ago	
	4-5 years ago	
	More than five years ago	
	Unsure/can't remember	

Question 8, ASK ALL, SC	Answer options ROWS	Tick one
Which of the following best describes the main environment where you provide care for cats within your organisation?	A cattery/shelter/rehoming centre (separate from a private domestic residence)	
	A **collection of pens** near to or adjoining a private residence (i.e. in a private garden or built as an extension to a private home)	
	A **single pen** near to or adjoining a private residence (i.e. in a private garden or built as an extension to a private home)	
	A room or rooms within a private domestic residence	
	Other<br>(Please provide some brief details)	OE, EXCLUSIVE

Question 9, ASK ALL, SC	Answer options ROWS	Tick one
Is this main place where you care for cats:	Part of a wider **national** organisation including multiple sites/catteries/branches/centres that care for cats across different regions	
	Part of a wider **local** organisation including multiple sites/catteries/centres that care for cats within a specific region	
	Independent (i.e. a single site) and **not part of a wider national or local organisation**	
	Other (Please provide some brief details)	OE
	Unsure/don't know	

Question 10, ASK ALL, SC	Answer options HEADINGS ROWS	Answer options	Tick one
Where is the place that you care for cats located?<br><br>(this information will be used to determine how representative our study sample is and will **not** be used to identify your individual charity/organisation)	England	North East	
		North West	
		Yorkshire and the Humber	
		West Midlands	
		East Midlands	
		East England	
		London	
		South East	
		South West	
	Wales	North	
		Mid/Central	
		South	
	Scotland	Highlands and Islands	
		Grampian	
		Lothian and Borders	
		Tayside	
		Fife	
		Strathclyde	
		Central Scotland	
		Dumfries and Galloway	
	Republic of Ireland	Connacht/Connaught	
		Leinster	
		Munster	
		Ulster	
	Northern Ireland		
	Isle of Man or UK Channel Islands (E.g. Guernsey, Jersey, Alderney, Herm and Sark)		
	Unsure/don't know		
	Prefer not to say		

Question 11 ASK ALL, MC	Answer options ROWS	Tick all that apply
What kinds of services are provided at this location?	Temporary care and housing of shelter/rescue cats and their subsequent rehoming to members of the public	
	Permanent care and housing of cats	
	Temporary care and housing of cats (for commercial purposes such as paid boarding for privately-owned cats)	
	Trap, neuter and return of unowned (i.e. stray/feral/street/community) cats	
	Trap, neuter and relocate of unowned cats	
	Neutering of owned cats within the community	
	Temporary care and housing of other species	
	Permanent care and housing of other species	
	Other<br>(Please provide some brief details)	OE

Question 12 ASK ALL, SC	Answer options ROWS	Tick one
Is your charity/organisation a member of the Association of Dogs and Cats Homes (ADCH)?<br><br>If you are unsure and want to check, a current list of ADCH members can be found here: ADCH-Members-List-September-2022.pdf	No	
	Yes	
	Unsure/don't know	
	Not applicable	

Section 2: Numbers of staff and volunteers at your location

INFO: SHOW ALL

This section took participants an average of 1 minute~~s~~ to complete.

P2 ASK ALL, FORCE RESPONSE

Would you like to:

- ☐ Answer these questions
- ☐ Skip these questions

SHOW ALL OF THESE INTRODUCTION TEXTS ON SAME PAGE AS SECTION 2

Thinking about the specific location (e.g. foster room/pen/site/cattery/centre) where you spend most time caring for cats…..

**Question 13, ASK ALL, OE NUMERIC**		**Text box answer options MAX VALUE - 1001**
How many people are paid staff members?<br>*If you don't know the exact number, please estimate.*	Full time staff:	**INSERT OE BOX**
		Unsure/don't know~~:~~**, SC**
	Part time staff:	**INSERT OE BOX**
		Unsure/don't know~~:~~**, SC**

**Question 14, ASK ALL, OE NUMERIC**	**Text box answer options MAX VALUE – 10,001**
How many are volunteers?<br>*If you don't know the exact number, please estimate.*	**INSERT OE BOX**
	Unsure/don't know~~:~~**, SC**

Section 3: Current numbers of cats, pens and cats per staff at your location

INFO: SHOW ALL

This section took participants an average of 2 minutes to complete.

P3 ASK ALL, FORCE RESPONSE

Would you like to:

- ☐ Answer these questions
- ☐ Skip these questions

Thinking about the specific location (e.g. foster room/pen/site/cattery/centre) where you spend most time caring for cats…..

PLEASE SHOW Q15 AND Q16 ON SAME PAGE

Question 15, ASK ALL, OE NUMERIC	Text box: min value – 1 MAX VALUE - 1001
How many cats (including any kittens) on average would a single person be responsible caring for on a typical day?<br>*If you don't know the exact number, please estimate.*	INSERT OE BOX
	Unsure/don't know SC

Question 16, ASK ALL, OE NUMERIC	Answer options MAX VALUE - 1001	Question 17<br>If added in any values to Q16= number values, Q17 to appear:<br><br>Of these cats currently in care, how many are:	Answer options: NUMBER CANNOT BE MORE THAN ANSWER AT Q16 BUT CAN BE LESS
How many cats are there in care at the moment?<br>*If you don't know the exact number, please estimate.*	INSERT OE BOX	Cats over 16 weeks of age	INSERT OE BOX
			Unsure/don't know SC
	Unsure/don't know: SC	Kittens 16 weeks of age or under	INSERT OE BOX
			Unsure/don't know: SC

Question 18, ASK ALL,OE NUMERIC	Text box options: min value – 1 MAX VALUE - 1001
How many separate units/pens/rooms/areas are there to house cats?<br>*If you don't know the exact number, please estimate.*	INSERT OE BOX
	There is only one unit/pen/room/area to house cats at this location SC
	Unsure/don't know: SC

Section 4: Cat length of stay, intake/admissions and waiting lists:

INFO: SHOW ALL

This section took participants an average of 4 minutes to complete.

P4 ASK ALL, FORCE RESPONSE

Would you like to:

- ☐ Answer these questions
- ☐ Skip these questions

Thinking about the specific location (e.g. foster room/pen/site/cattery/centre) where you spend most time caring for cats…..

Question 18a, ASK ALL, OE NUMERIC	MIN 0 DAYS, MAX 5001 DAYS
Over the past 12 months what is the **average** time from a cat entering your site/cattery/centre to it leaving?	
For cats that have been trapped, neutered and returned/relocated *If you don't know the exact number, please estimate*	OE
	Unsure/Don't know SC
	Not applicable SC
For cats that have been rehomed as pets/human companions *If you don't know the exact number, please estimate*	OE
	Unsure/Don't know SC
	Not applicable SC

Question 18b, ASK ALL, OE NUMERIC	MIN 0 DAYS, MAX 5001 DAYS
Over the past 12 months what is the **shortest** time from a cat entering your site/cattery/centre to it leaving?	
For cats that have been trapped, neutered and returned/relocated *If you don't know the exact number, please estimate*	OE
	Unsure/Don't know SC
	Not applicable SC
For cats that have been rehomed as pets/human companions *If you don't know the exact number, please estimate*	OE
	Unsure/Don't know SC
	Not applicable SC

Question 18c, ASK ALL, OE NUMERIC	MIN 0 DAYS, MAX 5001 DAYS
Over the past 12 months what is the **longest** time from a cat entering your site/cattery/centre to it leaving?	
For cats that have been trapped, neutered and returned/relocated *If you don't know the exact number, please estimate*	OE
	Unsure/Don't know SC
	Not applicable SC
For cats that have been rehomed as pets/human companions *If you don't know the exact number, please estimate*	OE
	Unsure/Don't know SC
	Not applicable SC

Question 19, ASK ALL, SC	Tick one
Is a waiting list used to manage enquiries for cats to be admitted/brought into care?	Yes
	No
	Unsure/don't know
[if answered 'yes' for Q19, q20 to appear on ~~same~~ separate screen:	INSERT OE BOX

Q20 OE NUMERIC MAX VALUE - 1001<br>How many cats are currently on the waiting list?<br>*If you don't know the exact number, please estimate.*	 Unsure/don't know: SC

Question header, SHOW ALL: Are any assessment methods or criteria used to:		
Q 21, ASK ALL, SC<br>Determine whether a cat is **suitable** to admit/bring into care or not?	Yes	Please provide some brief details: OE
	No	
	Unsure/don't know	
Q 22 , ASK ALL, SC<br>Determine the **priority level** of a cat to be admitted/brought into care?	Yes	Please provide some brief details: OE
	No	
	Unsure/don't know	

Question 23, ASK ALL, SC	Tick one
Are there units/pens/areas that are routinely kept empty and reserved for emergency admission/intake situations?	Never
	Sometimes or occasionally
	Usually
	Always
	Unsure/don't know

Question 24, ASK ALL, OE NUMERIC	Answer options ROWS	Text box options: MAX VALUE - 1001
From 1st January 2022-31st December 2022, how many cats were admitted?<br>*If you don't know the exact number, please estimate.*	Total number of cats	INSERT OE BOX<br><br>Unsure/don't know SC
	[if respondent enters a value for q24, q24.1-24.5 to appear] value cannot be more than Q24 but does not have to total to q24<br><br>If known, of these admitted cats, how many came in:	Text box options:
	**q24a.1** As an unowned cat (e.g. stray/feral/street/community cat/stray from vets or other charity/kittens born on site)	INSERT OE BOX<br><br>Unsure/don't know SC
	**q2 a 4.2** As a relinquished previously owned cat (e.g. relinquished by owner or next of kin/owned cat from vet surgery/other charity)	INSERT OE BOX<br><br>Unsure/don't know SC
	**q24 a.3** As an owned cat, removed from owner for welfare reasons	INSERT OE BOX<br><br>Unsure/don't know SC
	**q24 a.4** As an owned cat for which temporary care was provided (i.e. due to owner being unwell, escaping domestic abuse etc)	INSERT OE BOX<br><br>Unsure/don't know SC
	**q24 a.5** Other (i.e. cats that came in from sources not included in the above options)	INSERT OE BOX<br><br>Unsure/don't know SC

Question 25, ASK ALL, OE NUMERIC MAX VALUE - 1001	Answer options	Text box options:
Over the past 12 months, what is the greatest number of cats to have been housed at any one time?<br>*If you don't know the exact number, please estimate.*	Total number of cats	INSERT OE BOX<br><br>Unsure/don't know SC
	[if respondent enters a value above for q25, q25a.1-25.2 to appear] value must total Q25<br><br>**Q25a**<br>If known, how many of these cats were:	Text box options:
	Q24a.1<br>Cats over 16 weeks of age	INSERT OE BOX<br><br>Unsure/don't know SC
	Q24a.2<br>Kittens aged 16 weeks or under	INSERT OE BOX<br><br>Unsure/don't know SC

Section 5: Environmental provisions within cat pens/units:

INFO: SHOW ALL

This section took participants an average of 2 minutes to complete.

Thinking about the specific location (e.g. foster room/pen/site/cattery/centre) where you spend most time caring for cats…..

Question header, Q26 ASK ALL, SC PER ROW, GRID	ROWS
How often are cats typically provided with:	A hiding place at ground level
	An elevated surface such as a shelf
	A hiding place on an elevated area such as a shelf
	A litter tray that is located away from beds, food and water bowls
	An area with soft material such as a blanket or cat bed
	Puzzle feeding devices (i.e. home made or shop bought devices that encourage the cat to work/problem solve to obtain food)
	Toys
	Scratching opportunities
	A water bowl
	A food bowl
COLUMNS	
	Never
	Sometimes or occasionally
	Usually
	Always
	Unsure/don't know

Q26a ASK ALL WHO SELECTED SOMETIMES OR OCCASIONALLY–ALWAYS FOR Q26, SC PER ROW GRID<br>When providing this item in a cat's pen/unit, how many would typically be provided?	ROWS
	INSERT SELECTED AT Q26

COLUMNS	More than one
**For cats housed singly**	One
	Unsure/don't know
	Not applicable

Q26b COLUMNS	More than one per cat
**For cats housed together**	One per cat
	Less than one per cat
	Unsure/don't know
	Not applicable

Section 6: Moving of cats between different pens/units:

INFO: SHOW ALL

This section took participants an average of 2 minutes to complete.

P6 ASK ALL, FORCE RESPONSE

Would you like to:

- Answer these questions
- Skip these questions

Thinking about the specific location (e.g. foster room/pen/site/cattery/centre) where you spend most time caring for cats…..

**Q27** ASK ALL, SC PER ROW, GRID<br>Prior to being homed/adopted, how often do cats:	ROWS
	Stay at this location and stay in the same pen/unit/room
	Stay at this location but move between different pens/units/rooms
	Move to a different location within your charity/organisation (i.e. a different foster home/site/cattery/centre)
COLUMNS	Never
	Sometimes or occasionally
	Usually
	Always
	Unsure/don't know
	Not applicable

Q27a SHOW IF CODES SOMETIMES-ALWAYS AT ROWS 2 AND 3 AT Q27, OE NUMERIC	
SHOW FOR ROW 2 AT Q27	On average, how many times might a cat move between different pens or areas in your location?
	Unsure/Don't know
SHOW FOR ROW 3 AT Q27	On average, how many times might a cat move between different sites/catteries/centres?
	Unsure/Don't know

Section 7: Single and multicat housing:

INFO: SHOW ALL

This section took participants an average of 2 minutes to complete.

P7 ASK ALL, FORCE RESPONSE

Would you like to:

- ☐ Answer these questions
- ☐ Skip these questions

Thinking about the specific location (e.g. foster room/pen/site/cattery/centre) where you spend most time caring for cats…

Question 28, ASK ALL, OE	Text box: range 1-1001
Excluding nursing queens and kittens, what is the maximum number of cats that would be housed together in an individual pen/unit/room/area?<br>*If you don't know the exact number, please estimate.*	INSERT OE BOX
	Unsure/don't know SC

Question 29 ASK ALL, SC	
Are kittens from different litters ever mixed/housed together?	Never
	Sometimes or occasionally
	Usually
	Always
	Unsure/don't know

Question 30 ASK ALL, SC	Tick one
	Never
	Sometimes or occasionally
	Usually
	Always
At the location where you care for cats, how often would cats originating from **the same previous household/living environment** be housed together?	Unsure/don't know
Q31, SC, CAROUSEL ASK *If answered Sometimes or occasionally-always for q30: SC*	
ROWS	
	Never
	Sometimes or occasionally
	Usually
	Always
How often would cats originating from **the same previous environment** be separated if they did not appear to be **actively enjoying** each others company, (i.e. lack of mutual play, grooming, sleeping and resting together by choice) when housed together?	Unsure/don't know
	Never
	Sometimes or occasionally
	Usually
	Always
How often would cats originating from **the same previous environment** be separated if they appeared to be **actively struggling** in each others company, (i.e. fighting, resource guarding or blocking) when housed together?	Unsure/don't know
	Never
	Sometimes or occasionally
	Usually
	Always
How often would a cat's reported relationships with each other in their **previous shared environment** be used to determine whether to house them together or not at your charity/organisation?	Unsure/don't know

**Question 32**: **SC, ASK ALL**	**Tick one**
At the location where you care for cats, how often would cats originating from **different previous households/living environments** be housed together?	Never<br>Sometimes or occasionally<br>Usually<br>Always<br>Unsure/don't know
**Q33, SC, CAROUSEL ASK *If answered Sometimes or occasionally-always for q32:***	
How often would cats originating from **different previous environments** be separated if they did not appear to be **actively enjoying** each others company, (i.e. lack of mutual play, grooming, sleeping and resting together by choice) when housed together?	Never<br>Sometimes or occasionally<br>Usually<br>Always<br>Unsure/don't know
How often would cats originating from **different previous environments** be separated if they appeared to be **actively struggling** in each others company (i.e. fighting, resource guarding or blocking) when housed together?	Never<br>Sometimes or occasionally<br>Usually<br>Always<br>Unsure/don’t know
How often would a cat’s reported relationships with any other cat they might have encountered in their **previous living environment** be used to determine whether to house them with other unfamiliar cats at your charity/organisation or not?	Never<br>Sometimes or occasionally<br>Usually<br>Always<br>Unsure/don’t know

Section 8: Rough average sizes of single and multi-cat pens/units:

INFO: SHOW ALL

This section took participants an average of 2 minutes to complete.

P8 ASK ALL, FORCE RESPONSE

Would you like to:

- ☐ Answer these questions
- ☐ Skip these questions

Thinking about the specific location (e.g. foster room/pen/site/cattery/centre) where you spend most time caring for cats…..

Question 34 ASK ALL, OE NUMERIC	Text box: (options to enter in imperial) PLEASE ADD CM TO THE END OF EACH OE NUMERIC BOX<br>Note: please answer in terms of the rough **total area** the cat has access to, based on the measurements of the external perimeters of their individual enclosure<br>*If you are unsure, please estimate*
Question heading: **For cats over 16 weeks of age**	
Roughly, what would be the **typical dimensions** of a pen/unit/room/area a **single cat** would be housed in?	Length
	Width
	Height
	Unsure/don't know SC, EXCLUSIVE
	Not applicable SC, EXCLUSIVE
Roughly, what would be the **typical dimensions** of a pen/unit/room/area a **pair of cats** would be housed together in?	Length
	Width
	Height
	Unsure/don't know SC, EXCLUSIVE
	Not applicable SC, EXCLUSIVE
Roughly, what would be the **typical dimensions** of a pen/unit/room/area a **group of cats (i.e 3+)** would be housed together in?	Length
	Width
	Height
	Unsure/don't know SC, EXCLUSIVE
	Not applicable SC, EXCLUSIVE

Section 9: Preventative health care and vet checks:

INFO: SHOW ALL

This section took participants an average of 3 minutes to complete.

P9 ASK ALL, FORCE RESPONSE

Would you like to:

- ☐ Answer these questions
- ☐ Skip these questions

Thinking about the specific location (e.g. foster room/pen/site/cattery/centre) where you spend most time caring for cats…..

SHOW Q35, 36 AND 37 ON SAME PAGE

Question 35 ASK ALL, SC	Tick one
Are cats' parasite treatments kept up to date?	Never
	Sometimes or occasionally
	Usually
	Always
	Unsure/don't know
**Question 36 ASK ALL, SC**	**Tick one**
Are cats' vaccination courses kept up to date?	Never
	Sometimes or occasionally
	Usually
	Always
	Unsure/don't know

Question 37 ASK ALL, SC	Tick one
Are cats seen by a registered Veterinary Surgeon (RCVS) or Veterinary Nurse (RVN) for a routine health check/screening prior to homing?	Never
	Sometimes or occasionally
	Usually
	Always
	Unsure/don't know
**Question 38, OE, ASK if responded usually-never to Q37**	
What reasons might cats not be seen by a vet or nurse before being ~~re~~homed?	Open text box
	Unsure/Don't know
**Question 38b , OE, ASK if responded Sometimes or occasionally-always to Q37** What is the average time from a cat's arrival to them being seen by a vet or vet nurse for a routine health check/screening?	INSERT OE BOX WITH DAYS AS THE UNIT

If you don't know the exact number, please estimate.	Less than a day/Same day SC, EXCLUSIVE
	Unsure/don't know SC EXCLUSIVE

Question 39 ASK ALL, MC	Tick all that apply
How do cats usually receive any required veterinary examinations?	Cats are taken to a separate veterinary practice as and when needed
	Someone from a separate veterinary practice visits when requested
	Someone from a separate veterinary practice visits on set/regular days
	Cats receive veterinary examinations via resident vets and/or nurses that are based here
	Unsure/don't know EXCLUSIVE
	Other (Please provide some brief details) OE

Question 40 ASK ALL	Open text box:
Is there any formal guidance around when cat carers should seek veterinary advice for cats with a suspected or ongoing physical health condition?	Yes (Please provide some brief details) OE
	No guidance provided SC
	Unsure/don't know SC

Question 41 ASK ALL, SC	Tick one
If cats have a suspected or confirmed contagious disease, are there options for them to be placed in isolation facilities? (i.e. kept in a self contained pen/unit/room/area away from healthy cat populations)	Yes
	No
	Unsure/don't know

Question 42 ASK ALL, SC	Tick one
For a cat that is pregnant at the point of admission, would a pregnant spay/neutering procedure be undertaken to prevent the cat from having the litter?	Never
	Sometimes or occasionally
	Usually
	Always
	Unsure/don't know
	Not applicable

Section 10: Medicating cats and handling for health checks:

INFO: SHOW ALL.

This section took participants an average of 3 minutes to complete.

P10 ASK ALL, FORCE RESPONSE

Would you like to:

- ☐ Answer these questions
- ☐ Skip these questions

Thinking about the cats cared for at your specific location (e.g. foster room/pen/site/cattery/centre) …..

**Question** 44 ASK ALL, SC PER ROW, CAROUSEL		**Tick one** COLUMNS
During any **routine health checks** cats experience (i.e. those performed by either cat care staff or vets/nurses), how often are the following methods applied:	Cat restrained by using minimal touch or lightly holding the cat's body/head still with person's arms/hands	Never<br>Sometimes or occasionally<br>Usually<br>Always<br>Unsure/don't know
	Cat restrained by firmly holding the cat's body/head still with person's arms/hands	Never<br>Sometimes or occasionally<br>Usually<br>Always<br>Unsure/don't know
	Cat restrained using a towel wrapped around them	Never<br>Sometimes or occasionally<br>Usually<br>Always<br>Unsure/don't know
	Treats used to get the cat to remain still/to move into a desired position	Never<br>Sometimes or occasionally<br>Usually<br>Always<br>Unsure/don't know
	Cat restrained using a sedative	Never<br>Sometimes or occasionally<br>Usually<br>Always<br>Unsure/don't know
	Cat restrained using a carrier/cage with sliding sections that restrict the cat's movement	Never<br>Sometimes or occasionally<br>Usually<br>Always<br>Unsure/don't know
	Cat restrained by scruffing or applying a clip to the back of their neck	Never<br>Sometimes or occasionally<br>Usually<br>Always<br>Unsure/don't know
		Never<br>Sometimes or occasionally

	Cat restrained by using a thundershirt, muzzle, headcollar or similar equipment designed to restrict elements of the cat’s movement or behaviour	Usually<br>Always<br>Unsure/don't know

Question 43 ASK ALL, SC PER ROW, ~~GRID~~ CAROUSEL		Tick one COLUMNS
When **administering health treatments** to cats, how often are the following methods used:	Oral medication put into food and presented to the cat to consume	Never<br>Sometimes or occasionally<br>Usually<br>Always<br>Unsure/don't know
	Oral medication put into food and then directly placed in the cat's mouth	Never<br>Sometimes or occasionally<br>Usually<br>Always<br>Unsure/don't know
	Oral medication placed directly in the cat's mouth (without food)	Never<br>Sometimes or occasionally<br>Usually<br>Always<br>Unsure/don't know
	Oral medication administered directly in the cat's mouth using a ‘pill popper’ or similar device	Never<br>Sometimes or occasionally<br>Usually<br>Always<br>Unsure/don't know
	Cat restrained by using minimal touch or lightly holding the cats body/head still with person's arms/hands	Never<br>Sometimes or occasionally<br>Usually<br>Always<br>Unsure/don't know
	Cat restrained by firmly holding the cats body/head still with person's arms/hands	Never<br>Sometimes or occasionally<br>Usually<br>Always<br>Unsure/don't know
	Cat restrained using a towel wrapped around them	Never<br>Sometimes or occasionally<br>Usually<br>Always<br>Unsure/don't know
	Treats used to get the cat to remain still/to move into a desired position	Never<br>Sometimes or occasionally<br>Usually<br>Always<br>Unsure/don't know
	Cat restrained using a carrier/cage with sliding sections that restrict the cat’s movement	Never<br>Sometimes or occasionally<br>Usually<br>Always<br>Unsure/don't know
	Cat restrained by scruffing or applying a clip to the back of their neck	Never<br>Sometimes or occasionally

		Usually
		Always
		Unsure/don't know
	Cat restrained by using a thundershirt, muzzle, headcollar or similar equipment designed to restrict elements of the cat’s movement or behaviour	Never
		Sometimes or occasionally
		Usually
		Always
		Unsure/don't know

<u>Section 11: Cleaning cat pens/units</u>

INFO: SHOW ALL

This section took participants an average of 2 minutes to complete.

P11 ASK ALL, FORCE RESPONSE

Would you like to:

- ☐ Answer these questions
- ☐ Skip these questions

Thinking about the cats cared for at your specific location (e.g. foster room/pen/site/cattery/centre) …..

**Question 45 ASK ALL, SC PER ROW, CAROUSEL**		**Tick one COLUMNS**
For resident **healthy adult cats** (i.e. disease free), how often would:	The cat's pen be spot cleaned (i.e. only soiled items removed/dirty surfaces cleaned)	Once a month or less
		A few times a month
		Once or twice a week
		Every day or most days
		Unsure/don't know
	All/most vertical and horizontal surfaces within a cat's pen be fully cleaned with disinfectant	Once a month or less
		A few times a month
		Once or twice a week
		Every day or most days
		Unsure/don't know
	Any *non-soiled* soft furnishings (i.e. beds, blankets, towels) be removed and replaced with clean items	Once a month or less
		A few times a month
		Once or twice a week
		Every day or most days
		Unsure/don't know
	Existing litter tray swapped for a clean tray and fresh litter	Once a month or less
		A few times a month
		Once or twice a week
		Every day or most days
		Unsure/don't know
	Fresh food be provided in a clean bowl/tray and/or puzzle feeding devices	Once a month or less
		A few times a month
		Once or twice a week
		Every day or most days
		Unsure/don't know
	Fresh water be provided in a clean bowl	Once a month or less
		A few times a month
		Once or twice a week

		Every day or most days<br>Unsure/don't know

Question 46 ASK ALL, SC PER ROW, CAROUSEL		Tick one COLUMNS
When **undertaking cleaning** of pens/units/rooms/areas occupied by **healthy adult cats**, how often would you:	Physically move the cat from where they are currently located in their pen to another area of their pen to facilitate cleaning	Never<br>Sometimes or occasionally<br>Usually<br>Always<br>Unsure/don't know
	Physically place the cat into a carrier or another area outside of their pen to facilitate cleaning	Never<br>Sometimes or occasionally<br>Usually<br>Always<br>Unsure/don't know
	Leave or avoid cleaning certain areas so as not to disturb a cat (i.e. that is hiding/resting/sleeping)	Never<br>Sometimes or occasionally<br>Usually<br>Always<br>Unsure/don't know
	Use separate gloves in between pens	Never<br>Sometimes or occasionally<br>Usually<br>Always<br>Unsure/don't know
	Wash or sanitise hands in between pens	Never<br>Sometimes or occasionally<br>Usually<br>Always<br>Unsure/don't know
	Use separate protective clothing items (eg any or combination of aprons, overshoes, overalls or body suits) in between pens	Never<br>Sometimes or occasionally<br>Usually<br>Always<br>Unsure/don't know
	Use separate pen cleaning items/equipment in between pens	Never<br>Sometimes or occasionally<br>Usually<br>Always<br>Unsure/don't know
	Remove any *soiled* soft furnishings (ie beds, blankets, towels) and replace with clean items	Never<br>Sometimes or occasionally<br>Usually<br>Always<br>Unsure/don't know
	Removed any *soiled* litter from trays (and replace with fresh litter where needed)	Never<br>Sometimes or occasionally<br>Usually<br>Always<br>Unsure/don't know
		Never

	Clean litter trays away from food preparation areas or at times outside of food preparation times	Sometimes or occasionally
		Usually
		Always
		Unsure/don't know
	Fully clean, disinfect and dry out a pen and any items within it at change of occupancy	Never
		Sometimes or occasionally
		Usually
		Always
		Unsure/don't know

<u>Section 12: Cat socialisation, interactions and behaviour management</u>

INFO: SHOW ALL

This section took participants an average of 5 minutes to complete.

Thinking about the cats that are admitted to the location (e.g. foster room/pen/site/cattery/centre) where you care for cats…

Question 47 ASK ALL, SC PER ROW, ~~GRID~~ CAROUSEL		Tick one COLUMNS
~~:~~ In general, for **physically healthy** cats that appear unfriendly/fearful/behave aggressively, what approaches might be used to try to improve their behaviour and/or encourage them to accept human proximity/being stroked?	Approach and touch the cat while they are in a hiding area or an elevated position	Never
		Sometimes or occasionally
		Usually
		Always
		Unsure/don't know
	Move the cat from their current hiding area or elevated position for the purposes of interactions	Never
		Sometimes or occasionally
		Usually
		Always
		Unsure/don't know
	Hold the cat or pick them up	Never
		Sometimes or occasionally
		Usually
		Always
		Unsure/don't know
	Touch/stroke the cat with an object other than a person's hand such as a paintbrush, touching wand or stick	Never
		Sometimes or occasionally
		Usually
		Always
		Unsure/don't know
	Touch/stroke the cat while wearing gauntlets or other protective clothing to reduce human injury	Never
		Sometimes or occasionally
		Usually
		Always
		Unsure/don't know
	Place the cat into a carrier/dog crate or similar that is located in a busy, human-frequented area	Never
		Sometimes or occasionally
		Usually
		Always
		Unsure/don't know
	Allow the cat to approach and make the initial contact during interactions	Never
		Sometimes or occasionally
		Usually
		Always
		Unsure/don't know
	Allow the cat to end interactions when they choose by letting them walk or move away	Never
		Sometimes or occasionally
		Usually
		Always
		Unsure/don't know
	Allow the cat to avoid human proximity and interactions by maintaining a distance from where they are hiding or perching (if in an elevated position)	Never
		Sometimes or occasionally
		Usually

		Always
		Unsure/don't know
	Ignore the cat completely and wait until they actively start to approach humans to interact with them	Never
		Sometimes or occasionally
		Usually
		Always
		Unsure/don't know
	Take the cat out of their pen/unit/room and to another area for the purposes of interacting with them	Never
		Sometimes or occasionally
		Usually
		Always
		Unsure/don't know
		Not applicable
	Move the cat to a more suitable pen/unit/room in order to provide them with living quarters that are larger or in a quieter area	Never
		Sometimes or occasionally
		Usually
		Always
		Unsure/don't know
		Not applicable
	Move the cat to a more suitable location (e.g. from a centre into a foster home/from one centre to another)	Never
		Sometimes or occasionally
		Usually
		Always
		Unsure/don't know
		Not applicable
	Any other approaches regularly used	

Question 47a ASK ALL WHO SAID SOMETIMES OR OCCASIONALLY - ALWAYS AT ANY OTHER APPROACHES AT Q47, OE	INSERT OE BOX
Please provide details of any other approaches that might be used to improve the cat's behaviour towards humans.	
	Unsure/Don't know

Question 48 ASK ALL, SC PER ROW<br><br>**For cats that appear to be unsocialised to humans and are very fearful* on arrival~~:~~, how often would attempts be made to socialise such cats:**<br><br>***Fearful cats might be constantly or frequently hiding or attempting to hide and either completely freeze, try to flee or escape and/or hiss, growl, bite or swipe when approached or touched**	Tick one, COLUMNS
If they were **under 8 weeks** of age	Never
	Sometimes or occasionally<br>Usually<br>Always

	Unsure/don't know
	Not applicable
If they were **between 8 and 16 weeks** of age	Never
	Sometimes or occasionally<br>Usually<br>Always
	Unsure/don't know
	Not applicable
If they were over **16 weeks** of age	Never
	Sometimes or occasionally<br>Usually<br>Always
	Unsure/don't know
	Not applicable

**QUESTION 48a, OE, ASK ALL WHO answered Sometimes or occasionally – always for any of question 48**	
Please provide some brief details of what this socialisation process would involve, how long it would usually take and what criteria would be used to determine if it was successful or unsuccessful	**INSERT OE BOX**
	Unsure/don't know

**Question 49 ASK ALL WHO ANSWERED SOMETIMES OR OCCATIONALLY – ALWAYS AT Q48, SC PER ROW,**	**Tick one COLUMNS**
Are there any circumstances under which attempts to start socialising very fearful cats would **not be attempted?**	Yes (Please provide some brief details) **OE**
	No
	Not applicable
	Unsure/don't know

Section 13: Cat rehoming assessments and processes:

INFO: SHOW ALL

This section took participants an average of 4 minutes to complete.

P13 ASK ALL, FORCE RESPONSE

Would you like to:

- ☐ Answer these questions
- ☐ Skip these questions

Thinking about the specific location (e.g. foster room/pen/site/cattery/centre) where you spend most time caring for cats…..

**Question 50 ASK ALL, SC**	**Tick one**
Are any methods used to assess a prospective adopter's suitability to home a particular cat?	Yes (Please specify) **OE**
	No
	Unsure/don't know

**Question 51 ASK ALL, SC**	**Tick one**
Are any methods used to assess a cat's suitability to be homed as a pet/human companion?	Yes (Please specify) **OE**
	No
	Unsure/don't know

**Q51a ASK ALL WHO ANSWERED YES AT Q51, SC PER ROW CAROUSEL**<br>How often would assessments to determine a cat's suitability to be homed as a pet/human companion be undertaken on the following types of cats?	**ROWS**
	Previously owned cats (i.e. relinquished from domestic homes)
	Stray or community cats
	Feral cats or those suspected to be feral
	Cats that exhibit aggressive behaviour towards staff or volunteers while housed at your location
	Cats that exhibit fearful behaviour towards staff or volunteers while housed at your location
	Cats that appear to be experiencing high stress levels and generally failing to cope while housed at your location
	Cats that do not seem to actively enjoy interactions with staff or volunteers (i.e. they may tolerate interactions but tend not to solicit them)
	Previously owned cats where the person relinquishing the cat provides information suggesting the cat exhibited behaviour problems in the past home
**COLUMNS**	Never
	Sometimes or occasionally

	Usually
	Always
	Unsure/don't know
	Not applicable

Question 52 ASK ALL, SC		Tick one
Are any methods used to assess a cat's:	Suitability to be homed with other cats?	Yes (Please provide brief details of the specific methods used) OE
		No
		Unsure/don't know
	Suitability to be homed with dogs?	Yes (Please provide brief details of the specific methods used) OE
		No
		Unsure/don't know
	Suitability to certain domestic lifestyles (i.e. indoor versus outdoor, number of adults/children in a home, ages of children etc)?	Yes (Please provide brief details of the specific methods used) OE
		No
		Unsure/don't know

Question 53 ASK ALL, SC	Tick one
Are any post adoption follow ups undertaken?	Never
	Sometimes or occasionally
	Usually
	Always
	Unsure/don't know

Question 54 ASK ALL, SC PER ROW, CAROUSEL		Tick one COLUMNS
How often would any unsocialised and fearful cats coming into care be homed to an 'alternative outlet'* rather than to a domestic home as a 'pet'?<br><br>*An alternative outlet could mean the cat is returned to their site of origin, homed to a farm, stables, or a large domestic garden with outdoor food/shelter provided	If on arrival they were *under 8 weeks* of age	Never
		Sometimes or occasionally
		Usually
		Always
		Unsure/don't know
	If on arrival they were *between 8 and 16 weeks* of age	Never
		Sometimes or occasionally
		Usually
		Always
		Unsure/don't know
	If on arrival they were over *16 weeks* of age	Never
		Sometimes or occasionally
		Usually
		Always
		Unsure/don't know

Question 55 ASK ALL, SC PER ROW, CAROUSEL		Tick one COLUMNS
For cats that have **medical issues** which are not life threatening but would make them difficult to successfully home with a member of the public, how often might they:	Be homed to a staff member or volunteer	Never<br>Sometimes or occasionally<br>Usually<br>Always<br>Unsure/don't know
	Be kept at your location (i.e. foster room/pen/site/cattery/centre) permanently	Never<br>Sometimes or occasionally<br>Usually<br>Always<br>Unsure/don't know
	Be kept at your location indefinitely, until the right home is found for the cat	Never<br>Sometimes or occasionally<br>Usually<br>Always<br>Unsure/don't know
	Be kept at your location for a limited time, until the right home is found for the cat or their wellbeing deteriorates	Never<br>Sometimes or occasionally<br>Usually<br>Always<br>Unsure/don't know
	Be transferred to a different location (i.e. to a different fosterer/site/cattery/centre) within your organisation or even to a different organisation to improve their chances of finding a suitable home	Never<br>Sometimes or occasionally<br>Usually<br>Always<br>Unsure/don't know
	Be euthanised	Never<br>Sometimes or occasionally<br>Usually<br>Always<br>Unsure/don't know

Question 56 ASK ALL, SC PER ROW, CAROUSEL		Tick one COLUMNS
For cats that have **behavioural issues** which would make them difficult to successfully home with a member of the public, how often might they:	Be homed to a staff member or volunteer	Never<br>Sometimes or occasionally<br>Usually<br>Always<br>Unsure/don't know
	Be kept at your location (i.e. foster room/pen/site/cattery/centre) permanently	Never<br>Sometimes or occasionally<br>Usually<br>Always<br>Unsure/don't know
	Be kept at your location indefinitely, until the right home is found for the cat	Never<br>Sometimes or occasionally<br>Usually<br>Always<br>Unsure/don't know
	Be kept at your location for a limited time, until the right home is found for the cat, or their wellbeing deteriorates	Never<br>Sometimes or occasionally<br>Usually<br>Always<br>Unsure/don't know
	Be found an alternative home to a typical domestic environment or 'pet' home	Never<br>Sometimes or occasionally<br>Usually<br>Always<br>Unsure/don't know
	Be transferred to a different location (i.e. to a different fosterer/site/cattery/centre) within your organisation, or even to a different organisation to improve their chances of finding a suitable home	Never<br>Sometimes or occasionally<br>Usually<br>Always<br>Unsure/don't know
	Be euthanised	Never<br>Sometimes or occasionally<br>Usually<br>Always<br>Unsure/don't know

Section 14: Assessing and managing cat's wellbeing:

INFO: SHOW ALL

This section took participants an average of 5 minutes to complete.

P14 ASK ALL, FORCE RESPONSE

Would you like to:

- ☐ Answer these questions
- ☐ Skip these questions

Thinking about the specific location (e.g. foster room/pen/site/cattery/centre) where you spend most time caring for cats…..

Question 57 ASK ALL, SC PER ROW, CAROUSEL		Tick one COLUMNS
How often do you feel you are able to get useful/reliable information about cats coming in to care that helps you know how to meet their individual needs:	From people bringing in *owned/relinquished cats*	Never
		Sometimes or occasionally
		Usually
		Always
		Unsure/don't know
	From people bringing in *unowned cats* (i.e. stray/feral/street/community)	Never
		Sometimes or occasionally
		Usually
		Always
		Unsure/don't know

Question 58 ASK ALL, SC	Tick one
Over the past 12 months or so, on average, how often would you say cats (including kittens) that come into care appear very fearful* (ie fearful for more than 48 hours post arrival) of people~~?~~	Never
	Once a month or less
	A few times a month
*A cat that is fearful in people's presence might be constantly or frequently hiding or attempting to hide, appear frozen, try to flee or escape and/or hiss, growl, bite or swipe when approached or touched)	Once or twice a week
	Every day or most days
	Unsure/don't know

Question 59 ASK ALL, SC	Tick one
Excluding vet/nurse checks, are any methods used to assess individual cats' wellbeing~~?~~ (ie their general physical and/or mental state)?	Yes (please specify) OE
	No
	Unsure/don't know
[if answered *yes* for Q59] SC	Tick one
Q59a How often would a cat's wellbeing typically be assessed using these methods?	Once a month or less
	A few times a month
	Once or twice a week
	Every day or most days
	Unsure/don't know

Question 60 ASK ALL, SC PER ROW, ~~GRID~~ CAROUSEL		Tick one COLUMNS
How often do **owned/relinquished** cats that come in to care:	Appear to be in a poor/compromised state of *physical* health at the *point of admission?*	Never
		Sometimes or occasionally
		Usually
		Always
		Unsure/don't know
	Appear to be in a poor/compromised state of *physical* health *two weeks post admission?*	Never
		Sometimes or occasionally
		Usually
		Always
		Unsure/don't know
	Appear to be in a poor/compromised state of *mental* health at the *point of admission?*	Never
		Sometimes or occasionally
		Usually
		Always
		Unsure/don't know
	Appear to be in a poor/compromised state of *mental* health *two weeks post admission?*	Never
		Sometimes or occasionally
		Usually
		Always
		Unsure/don't know

Question 61 ASK ALL, SC PER ROW, ~~GRID~~ CAROUSEL		Tick one COLUMNS
How often do **unowned cats** (i.e. stray/feral/street/community cats) that come in to care:	Appear to be in a poor/compromised state of *physical* health at the *point of admission?*	Never
		Sometimes or occasionally
		Usually
		Always
		Unsure/don't know
	Appear to be in a poor/compromised state of *physical* health *two weeks post admission?*	Never
		Sometimes or occasionally
		Usually
		Always
		Unsure/don't know
	Appear to be in a poor/compromised state of *mental* health at the *point of admission?*	Never
		Sometimes or occasionally
		Usually
		Always
		Unsure/don't know
	Appear to be in a poor/compromised state of *mental* health *two weeks post admission?*	Never
		Sometimes or occasionally
		Usually
		Always
		Unsure/don't know

Question 62 ASK ALL, SC	Tick one:	
If a cat was deemed to be in a **poor/compromised mental state** (e.g. experiencing stress, anxiety, fear, frustration) while in care, would you apply any interventions to try to improve/resolve this?	Yes (please specify) OE	
	Not applicable/No interventions would be applied	
	Unsure/don't know	
Q62a [If answered 'yes' to q62, to appear]	CAROUSEL	COLUMNS
Before applying any of these interventions, how often would advice typically be sought from any of the following people?	A Registered Veterinary Nurse (RVN)	Never
		Sometimes or occasionally
		Usually
		Always
		Unsure/don't know
	A Registered Veterinary Surgeon (RCVS)	Never
		Sometimes or occasionally
		Usually
		Always
		Unsure/don't know
	A senior staff member (~~e.g.~~ eg centre manager)	Never
		Sometimes or occasionally
		Usually
		Always
		Unsure/don't know
	A more experienced staff member (eg has worked at your organisation for longer)	Never
		Sometimes or occasionally
		Usually
		Always
		Unsure/don't know
	A senior or more experienced volunteer (~~e.g.~~ eg has volunteered at your organisation for longer)	Never
		Sometimes or occasionally
		Usually
		Always
		Unsure/don't know
	A staff member or volunteer with training/qualifications in animal behaviour/welfare	Never
		Sometimes or occasionally
		Usually
		Always
		Unsure/don't know
	An external professional with qualifications in animal behaviour	Never
		Sometimes or occasionally
		Usually
		Always
		Unsure/don't know

Question 63, ASK ALL OE	Tick/Open text box:
If a cat is deemed to be in a **poor/compromised mental and physical state** while in care, are there any specific circumstances under which you might consider euthanising the cat to be a suitable outcome?	Yes (Please provide some brief details)
	No SC
	Not applicable
	Unsure/don't know SC

Question, 64 ASK ALL OE	Tick/Open text box:
If a cat is deemed to be in a **good mental and physical state** while in care, are there any specific circumstances under which you might consider euthanising the cat to be a suitable outcome?	Yes (Please provide some brief details)
	No SC
	Not applicable
	Unsure/don't know SC

Question, 65 ASK ALL SC	Tick one
Does your charity/organisation have any specific policies or guidance on euthanasia?	Yes (please provide some brief details) OE
	No
	Unsure/don't know

Question 66 ASK ALL SC PER ROW, CAROUSEL		Tick one COLUMNS
At the location where you care for cats, over the past 12 months, have you felt any of the following resource limitations have caused compromises to the level of care provided to admitted cats?	A lack of sufficient physical space to house these cats comfortably	Never
		Sometimes or occasionally
		Usually
		Always
		Unsure/don't know
	A lack of sufficient financial resources to care for these cats comfortably	Never
		Sometimes or occasionally
		Usually
		Always
		Unsure/don't know
	A lack of sufficient staff and/or volunteers to care for these cats comfortably	Never
		Sometimes or occasionally
		Usually
		Always
		Unsure/don't know
	A lack of sufficient knowledge, experience or skills of caregivers to care for these cats comfortably	Never
		Sometimes or occasionally
		Usually
		Always
		Unsure/don't know
		Never
		Sometimes or occasionally

	A lack of sufficient access to veterinary practices/veterinary professionals to care for these cats comfortably	Usually
		Always
		Unsure/don't know
	A lack of suitable/appropriate homes for these cats to be rehomed to, or a lack of interest from suitable homes	Never
		Sometimes or occasionally
		Usually
		Always
		Unsure/don't know

Section 15: Your thoughts on Cat wellbeing tools and necessary support

INFO: SHOW ALL

This section took participants an average of 4 minutes to complete.

**Question 67 ASK ALL SC**	**Open text box:**
For cats within rehoming/rescue/shelter/sanctuary organisations across the **UK/British Isles**, do you think there are any current barriers to achieving optimal cat wellbeing (i.e. good mental and physical states)?	Yes (please specify) **OE**
	No
	Unsure/don't know
**Question 68 ASK ALL SC** Do you think there are any additional resources (e.g. staff/volunteers, expertise, training, funds, additional pens/housing, equipment and tools) that could help to improve the current situation?	Yes
	No
	Unsure/don't know

**Question 69 ASK ALL SC**	**Open text box:**
For the cats coming under the care of **your charity/organisation,** do you think there are any current barriers to achieving optimal cat wellbeing (i.e. good mental and physical states)?	Yes (please specify) **OE**
	No
	Unsure/don't know
**Question 70 ASK ALL SC** Do you think there are any additional resources (e.g. staff/volunteers, expertise, training, funds, additional pens/housing, equipment and tools) that could help to improve the current situation for **your charity/organisation**?	Yes (please specify) **OE**
	No
	Unsure/don't know

Imagine a new validated, non-invasive tool has been developed to help monitor and improve the wellbeing of cats in shelters. The format of the 'tool' would be designed based on the identified needs and preferences of cat caregivers, but as an example, using the tool might require caregivers to make practical observations of cats, and potentially engage with a checklist or flowchart and answer a set of questions. This information would then be used to support cat caregivers in practically monitoring, understanding and improving the wellbeing of cats whilst while in care.

**Question 71 ASK ALL SC PER ROW, GRID**		**Tick one COLUMNS**
How important do you feel it would be for the tool to be applied to the following cats:	All cats within the care of your site/centre/cattery	Very important
		Somewhat important
		Neutral
		Not important
		Unsure/don't know
	Cats where there are already concerns about their wellbeing (i.e. physical and/or mental state)	Very important
		Somewhat important
		Neutral
		Not important
		Unsure/don't know
	Cats that you might consider to be most 'at risk' of experiencing poor/compromised wellbeing, based on their intake information, previous history or lifestyle	Very important
		Somewhat important
		Neutral

		Not important<br>Unsure/don't know
	I wouldn't want to apply a wellbeing assessment tool to any cats	EXCLUSIVE

Question 72 ASK ALL SC PER ROW, GRID		Tick one COLUMNS
How important do you feel it would be for the developed tool to assess:	The **physical health** of cats (i.e. the current physical and physiological state of the cat and the presence/absence of basic health conditions and disease)	Very important<br>Somewhat important<br>Neutral<br>Not important<br>Unsure/don't know
	The **mental health** of cats (i.e. their current mental state and the presence/absence of positive and negative emotions)	Very important<br>Somewhat important<br>Neutral<br>Not important<br>Unsure/don't know
	The **physical and mental health** of cats	Very important<br>Somewhat important<br>Neutral<br>Not important<br>Unsure/don't know
	The **quality of life** of cats (i.e. their current overall experience of their life which considers all facets of their physical and mental health, in addition to their social and physical environment and the cat's interactions and engagements with these aspects)	Very important<br>Somewhat important<br>Neutral<br>Not important<br>Unsure/don't know
	I wouldn't want to use a wellbeing assessment tool on any cats	EXCLUSIVE

Question 73 ASK ALL SC PER ROW, GRID		Tick one COLUMNS
How important do you feel it would be for the tool to support you to:	Prevent **future compromises** to the physical and/or mental state of a **group of cats**	Very important<br>Somewhat important<br>Neutral<br>Not important<br>Unsure/don't know
	Prevent **future compromises** to the physical and/or mental state of an **individual cat**	Very important<br>Somewhat important<br>Neutral<br>Not important<br>Unsure/don't know
	**Improve the current** physical and/or mental state of a **group of cats**	Very important<br>Somewhat important<br>Neutral<br>Not important<br>Unsure/don't know
	**Improve the current** physical and/or mental state of an **individual cat**	Very important<br>Somewhat important<br>Neutral<br>Not important<br>Unsure/don't know
	Make **end-of-life** decisions for individual cats where euthanasia is being considered to end current or prevent future suffering	Very important<br>Somewhat important<br>Neutral<br>Not important

		Unsure/don't know
	I wouldn't want the tool to tell me anything/wouldn't want to apply a tool to any cats	**EXCLUSIVE**

If the tool was developed following the preferences you indicated above, and made available for your charity/organisation to use	
**Question 74 ASK ALL SC**	**Tick one**
Would you have any concerns about using this sort of 'tool' at the location where you care for cats?	Yes (Please provide details) **OE**
	No concerns

Thinking about generally using and applying the 'tool' and when practically collecting/recording information to support it's use:		
**Question 76 ASK ALL MC**	**Tick any that apply**	
Would you have any concerns about engaging with the following formats?	Paper forms	
	A hard copy of a manual	
	A digital copy of a manual	
	An app (either on a phone or other mobile device)	
	A website	
	A mobile phone	
	A tablet iPad or other digital mobile device other than a phone	
	A laptop	
	A desktop computer	
	No concerns	**EXCLUSIVE, SC**
	Unsure/don't know	**EXCLUSIVE, SC**

**Question 76a ASK ALL WHO CODE 1-9 AT Q76, OE**	**Open text box:**
Please let us know what your concerns would be about engaging with those formats?	
	Unsure/Don't know

**Question 77 ASK ALL OE**	**Open text box:**
Are there any other comments you would like to make about the survey and/or about the wellbeing of cats within the sector?	
	None **SC**

Section 16: Your demographic details

This final section asks some questions about you (you will not be asked to provide any identifying details about yourself).

This section took participants an average of 2 minutes to complete.

Question 78 ASK ALL SC	Answer options	Tick one
Please indicate your age	18-24	
	25-34	
	35-44	
	45-54	
	55-64	
	65-74	
	75-84	
	85+	
	Prefer not to say	

Question 79 ASK ALL OE	Open text box max value = 90
Please indicate the number of active years in total you have either worked and/or volunteered within the rehoming/shelter/rescue sector<br>*Please answer to the nearest whole year*	Don't know/Can't remember SC

Question 80 ASK ALL OE	Answer options	Open text box
How many of your **own cats** currently live with you?	Kittens aged 16 weeks or under	
	Cats over 16 weeks of age	
	I don't currently live with any cats or kittens	

Question 81 ASK ALL SC	Answer options
Do you hold any formal qualifications in animal behaviour, welfare, health or animal training?	Yes (Please specify) OE
	No
	Prefer not to say

Survey submission page:

P17 ASK ALL, FORCE RESPONSE

You have now completed all sections of the survey. Below is a list of questions that you previously chose to skip. Would you like to answer any of these questions now?

- ☐ Yes
- ☐ No, I'm ready to submit my survey

Before you submit your completed survey, please indicate your preferences for future contact in relation to this research project and also preferences for free gifts and prizes

- ☐ If I am within the first 700 people to submit a survey, I am happy to be contacted via the email address I provide on the next page in order to receive my Pets at Home £20 voucher*~~.~~
- ☐ I would like to be entered into the prize draw~~er~~ for the chance to win an ADCH 2024 conference place (one available) or an International Cat Care online course~~s~~ (11 available) *
- ☐ I would like to receive updates about the results of this survey and any publications
- ☐ I am happy to receive information about opportunities to get involved in future aspects of this research project
- ☐ None of these SKIP TO SUBMISSION PAGE

* The issuing of the vouchers and the prize draw for eligible entries will take place shortly after the survey closes. Successful participants will be contacted via the provided email address to confirm course preferences/issuing of vouchers.

Please enter an email address so we can contact you in relation to the preferences you specified on the previous page. Once submitted, your survey data will no longer be linked to any email address you have provided here.<br><br>Email address:

Please now submit your survey

- ☐ Submit survey

Post submission page

Thank you so much for the time you have given to complete this survey, it is greatly appreciated. The anonymised data participants have provided from this survey will be used to help develop and validate future practical cat wellbeing assessment and support tools for the sector.

If you have any questions or concerns about this research or the survey, please direct them to Cats Protection Feline Welfare research team: sheltersurvey@cats.org.uk

The following is a list of websites that you may find useful:

Home - Find A Vet (rcvs.org.uk)

Practitioners – ABTC

Unowned cats | International Cat Care (icatcare.org)

Home | Association of Dogs and Cats Homes | Supporting Animal Rescue (adch.org.uk)
