# Supplementary material for: Part 1: A Sector-Wide Survey of UK/British Isles Shelter Organisations Caring for Cats: Caregiver-Reported Approaches to Housing, Husbandry and General Care Provision
Source: Vet Sci. 2026 Jun 16;13(6):587. doi: 10.3390/vetsci13060587 (PMC13308460; doi:10.3390/vetsci13060587)
Supplement: Supplementary file 1 [file vetsci-13-00587-s001.zip › Document S2 - Survey Results v03.pdf]

Document S2: Survey Results

Survey responses by section

Section	n	Percent
Section 1: Details of the charities/organisations and participants roles	393	100%
Section 2: Numbers of staff and volunteers at your location	345	87.8%
Section 3: Numbers of cats, pens and cats per staff at the site	347	88.3%
Section 4: Cat intake/admissions and waiting lists	337	85.8%
Section 5: Environmental provisions within cat pens/units	393	100%
Section 6: Moving of cats between different pens/units	344	87.5%
Section 7: Details about single and multi-cat housings	364	92.6%
Section 8: Average sizes of single and multi-cat pens/units	290	73.1%
Section 9: Preventive health care and vet checks	368	93.6%
Section 10: Medicating cats and handling for health checks	346	88.3%
Section 11: Cleaning cat pens/units	361	91.9%
Section 12: Cat socialisation, interactions and behaviour management	393	100%
Section 13: Cat rehoming assessments and processes	332	84.5%
Section 14: Assessing and managing cat wellbeing	333	84.7%
Section 15: Participants' perspective on cat wellbeing tools and necessary support	393	100%
Section 16: Participant demographics	393	100%

Q12. Is your charity/organisation a member of the Association of Dogs and Cats Homes (ADCH)?

Response	n	Percent
Yes	268	68.2%
No	71	18.1%

Unsure/don't know	54	13.7%

Note: Percentages are based on 393 non-missing responses.

Q10. Where is the place that you care for cats located?

		Reported location of the respondent's site		Location of all identified/contacted cat shelter organisations/ individual branches and centres across the British Isles	
Response		n	Percent	n	Percent
England	North East	15	3.8%	39	3.6%
	North West	33	8.4%	118	11.0%
	Yorkshire and the Humber	26	6.6%	82	7.6%
	West Midlands	20	5.1%	93	8.7%
	East Midlands	29	7.4%	81	7.6%
	East England	40	10.2%	120	11.2%
	London	47	12.0%	52	4.9%
	South East	69	17.6%	165	15.4%
	South West	59	15.0%	103	9.6%
Wales	North	9	2.3%	15	1.4%
	Mid/Central	2	0.5%	10	0.9%
	South	8	2.0%	34	3.2%

Scotland	Highlands and Islands	3	0.8%	15	1.4%
	Grampian	3	0.8%	8	0.7%
	Lothian and Borders	2	0.5%	8	0.7%
	Tayside	3	0.8%	5	0.5%
	Fife	0	0.0%	7	0.7%
	Strathclyde	3	0.8%	15	1.4%
	Central Scotland	3	0.8%	7	0.7%
	Dumfries and Galloway	0	0.0%	3	0.3%
Republic of Ireland	Connacht/ Connaught	2	0.5%	11	1.0%
	Leinster	2	0.5%	34	3.2%
	Munster	0	0.0%	14	1.3%
	Ulster	0	0.0%	5	0.5%
Northern Ireland		12	3.1%	21	2.0%
Isle of Man or UK Channel Islands (E.g. Guernsey, Jersey, Alderney, Herm and Sark)		3	0.8%	7	0.7%
Unsure/don’t know		0	0.0%	0	0.0%
Prefer not to say		0	0.0%	0	0.0%

Note: Percentages are based on 393 non-missing responses.

Q8. Which of the following best describes the main environment where you provide care for cats within your organisation?

Response	n	Percent
A cattery/shelter/rehoming centre (separate from a private domestic residence)	222	56.5%
A **collection of pens** near to or adjoining a private residence (i.e. in a private garden or built as an extension to a private home)	49	12.5%
A **single pen** near to or adjoining a private residence (i.e. in a private garden or built as an extension to a private home)	27	6.9%
A room or rooms within a private domestic residence	90	22.9%
Other<br>(Please provide some brief details)	5	1.3%

Note: Percentages are based on 393 non-missing responses.

Q11. What kinds of services are provided at this location?

Response	n	Percent
Temporary care and housing of shelter/rescue cats and their subsequent rehoming to members of the public	383	97.5%
Permanent care and housing of cats	51	13.0%
Temporary care and housing of cats (for commercial purposes such as paid boarding for privately-owned cats)	16	4.1%
Trap neuter return of unowned (i.e. stray/feral/street/community) cats	167	42.5%

Trap neuter relocate of unowned cats	138	35.1%
Neutering of owned cats within the community	125	31.8%
Temporary care and housing of other species	104	26.5%
Permanent care and housing of other species	23	5.9%

Note: Percentages are based on 393 respondents. Multiple selections allowed.

Q28. Excluding nursing queens and kittens, what is the maximum number of cats that would be housed together in an individual pen/unit/room/area? If you don’t know the exact number, please estimate.

Response	n	Percent
1	23	6.32%
2	221	60.71%
3	61	16.76%
4	19	5.22%
5	8	2.20%
6	10	2.75%
7	0	0.0%
8	0	0.0%
9	0	0.0%
10	2	0.55%
Unsure/don’t know	20	5.49%

Note: Percentages are based on 364 non-missing responses.

Q30. At the location where you care for cats, how often would cats originating from the same previous household/living environment be housed together?

Response	n	Percent
Never	3	0.8%
Sometimes or occasionally	111	30.5%
Usually	214	58.8%
Always	32	8.8%
Unsure/don't know	4	1.1%

Note: Percentages are based on 364 non-missing responses.

Q31. How often would cats reported relationships with each other in their previous shared environment be used to determine whether to house them together or not at your charity/organisation?

Response	n	Percent
Never	4	1.1%
Sometimes or occasionally	54	15.1%
Usually	133	37.3%
Always	152	42.6%
Unsure/don't know	14	3.9%

Note: Percentages are based on 357 non-missing responses.

Q31. How often would cats originating from the same previous environment be separated if they did not appear to be actively enjoying each other’s company, (i.e. lack of mutual play, grooming, sleeping and resting together by choice) when housed together?

Response	n	Percent
Never	11	3.1%
Sometimes or occasionally	75	21%
Usually	129	36.1%
Always	135	37.8%

Response	n	Percent
Unsure/don't know	7	2%

Note: Percentages are based on 357 non-missing responses.

Q31. How often would cats originating from the same previous environment be separated if they appeared to be actively struggling in each other's company, (i.e. fighting, resource guarding or blocking) when housed together?

Response	n	Percent
Never	6	1.7%
Sometimes or occasionally	15	4.2%
Usually	42	11.8%
Always	290	81.2%
Unsure/don't know	4	1.1%

Note: Percentages are based on 357 non-missing responses.

Q32. At the location where you care for cats, how often would cats originating from different previous household/living environments be housed together?

Response	n	Percent
Never	278	76.4%
Sometimes or occasionally	55	15.1%
Usually	22	6%
Always	1	0.3%
Unsure/don't know	8	2.2%

Note: Percentages are based on 364 non-missing responses.

Q33. How often would a cat's reported relationships with any other cat they might have encountered in their previous home/living environment be used to determine whether to house them with other unfamiliar cats at your charity/organisation or not?

Response	n	Percent
Never	4	5.1%
Sometimes or occasionally	16	20.5%
Usually	21	26.9%
Always	33	42.3%
Unsure/don't know	4	5.1%

Note: Percentages are based on 78 non-missing responses.

Q33. How often would cats originating from different previous environments be separated if they did not appear to be actively enjoying each other's company, (i.e. lack of mutual play, grooming, sleeping and resting together by choice) when housed together?

Response	n	Percent
Never	3	3.8%
Sometimes or occasionally	12	15.4%
Usually	22	28.2%
Always	40	51.3%
Unsure/don't know	1	1.3%

Note: Percentages are based on 78 non-missing responses.

Q33. How often would cats originating from different previous environments be separated if they appeared to be actively struggling in each other's company, (i.e. fighting, resource guarding or blocking) when housed together?

Response	n	Percent
Never	3	3.8%
Sometimes or occasionally	4	5.1%
Usually	8	10.3%
Always	63	80.8%

Response	n	Percent
Unsure/don't know	0	0%

Note: Percentages are based on 78 non-missing responses.

Q34. Roughly, what would be the typical dimensions of a pen/unit/room/area a single cat would be housed in?

Dimension (m)	Response	n	Percent
Length	1 - 2	14	4.96%
	2 - 3	87	30.85%
	4 - 5	53	18.79%
	6 - 7	3	1.06%
	8 - 9	2	0.71%
	10 - 11	2	0.71%
	12 - 13	1	0.35%
	20 - 21	1	0.35%
	Unsure/don't know	119	42.20%
Width	1 - 2	104	36.88%
	2 - 3	47	16.67%
	4 - 5	10	3.55%
	6 - 7	2	0.71%
	Unsure/don't know	119	42.20%
Height	1	26	9.22%
	2 - 3	122	43.26%
	4 - 5	10	3.55%
	6 - 7	2	0.71%
	8 - 9	1	0.35%

Dimension (m)	Response	n	Percent
	10 - 11	2	0.71%
	Unsure/don't know	119	42.20%

Note: Percentages are based on 282 non-missing responses.

Q34. Roughly, what would be the typical dimensions of a pen/unit/room/area a pair of cats would be housed together in?

Dimension (m)	Response	n	Percent
Length	1	10	3.65%
	2 - 3	78	28.47%
	4 - 5	49	17.88%
	6 - 7	7	2.55%
	8 - 9	3	1.09%
	10 - 11	3	1.09%
	20 - 21	2	0.73%
	Unsure/don't know	122	44.53%
Width	1	83	30.29%
	2 - 3	51	18.61%
	4 - 5	12	4.38%
	6 - 7	3	1.09%
	8 - 9	2	0.73%
	10 - 11	1	0.36%
	Unsure/don't know	122	44.53%
Height	1	23	8.39%
	2 - 3	116	42.34%
	4 - 5	9	3.28%

Dimension (m)	Response	n	Percent
	6 - 7	2	0.73%
	8 - 9	1	0.36%
	10 - 11	1	0.36%
	Unsure/don't know	122	44.53%

Note: Percentages are based on 274 non-missing responses.

Q34. Roughly, what would be the typical dimensions of a pen/unit/room/area a group of cats (i.e., 3+) would be housed together in?

Dimension (m)	Response	n	Percent
Length	1	5	2.53%
	2 - 3	37	18.69%
	4 - 5	27	13.64%
	6 - 7	7	3.54%
	8 - 9	4	2.02%
	10 - 11	3	1.52%
	16 - 17	1	0.51%
	20 - 21	1	0.51%
	Unsure/don't know	113	57.07%
Width	1	28	14.14%
	2 - 3	37	18.69%
	4 - 5	14	7.07%
	6 - 7	3	1.52%
	8 - 9	1	0.51%
	10 - 11	2	1.01%
	Unsure/don't know	113	57.07%

Dimension (m)	Response	n	Percent
Height	1	11	5.56%
	2 - 3	65	32.83%
	4 - 5	6	3.03%
	6 - 7	1	0.51%
	10 - 11	1	0.51%
	14 - 15	1	0.51%
	Unsure/don't know	113	57.07%

Note: Percentages are based on 198 non-missing responses.

Q26. How often are cats typically provided with:

Resource	Response	n	Percent
A hiding place at ground level	Never	0	0.0%
	Sometimes or occasionally	23	5.9%
	Usually	28	7.1%
	Always	341	86.8%
	Unsure/don't know	1	0.3%
An elevated surface such as a shelf	Never	3	0.8%
	Sometimes or occasionally	9	2.3%
	Usually	28	7.1%
	Always	352	89.6%
	Unsure/don't know	1	0.3%
A hiding place on an elevated area such as a shelf	Never	7	1.8%
	Sometimes or occasionally	25	6.4%
	Usually	47	12%

Resource	Response	n	Percent
	Always	313	79.6%
	Unsure/don't know	1	0.3%
A litter tray that is located away from beds, food and water bowls	Never	3	0.8%
	Sometimes or occasionally	8	2%
	Usually	29	7.4%
	Always	353	89.8%
	Unsure/don't know	0	0.0%
An area with soft material such as a blanket or cat bed	Never	1	0.3%
	Sometimes or occasionally	0	0.0%
	Usually	5	1.3%
	Always	387	98.5%
	Unsure/don't know	0	0.0%
Puzzle feeding devices (i.e. home made or shop bought devices that encourage the cat to work/problem solve to obtain food)	Never	17	4.3%
	Sometimes or occasionally	157	39.9%
	Usually	100	25.4%
	Always	115	29.3%
	Unsure/don't know	4	1%
Toys	Never	0	0.0%
	Sometimes or occasionally	5	1.3%
	Usually	18	4.6%
	Always	370	94.1%
	Unsure/don't know	0	0.0%

Resource	Response	n	Percent
Scratching opportunities	Never	0	0.0%
	Sometimes or occasionally	9	2.3%
	Usually	23	5.9%
	Always	361	91.9%
	Unsure/don't know	0	0.0%
A water bowl	Never	0	0.0%
	Sometimes or occasionally	0	0.0%
	Usually	0	0.0%
	Always	393	100%
	Unsure/don't know	0	0.0%
A food bowl	Never	0	0.0%
	Sometimes or occasionally	1	0.3%
	Usually	1	0.3%
	Always	391	99.5%
	Unsure/don't know	0	0.0%

Note: Percentages are based on 393 non-missing responses.

Q26. When providing this item in a cat's pen/unit, how many would typically be provided for cats housed singly?

Resource	Response	n	Percent
A hiding place at ground level	More than one	211	53.8%
	One	162	41.3%
	Unsure/don't know	8	2.0%
	Not applicable	11	2.8%
An elevated surface such as a shelf	More than one	222	57.1%

Resource	Response	n	Percent
	One	151	38.8%
	Unsure/don't know	8	2.1%
	Not applicable	8	2.1%
A hiding place on an elevated area such as a shelf	More than one	148	38.4%
	One	211	54.8%
	Unsure/don't know	15	3.9%
	Not applicable	11	2.9%
A litter tray that is located away from beds, food and water bowls	More than one	163	41.8%
	One	216	55.4%
	Unsure/don't know	6	1.5%
	Not applicable	5	1.3%
An area with soft material such as a blanket or cat bed	More than one	295	75.3%
	One	89	22.7%
	Unsure/don't know	4	1.0%
	Not applicable	4	1.0%
Puzzle feeding devices	More than one	84	22.6%
	One	215	57.8%
	Unsure/don't know	47	12.6%
	Not applicable	26	7.0%
Toys	More than one	365	92.9%
	One	15	3.8%
	Unsure/don't know	7	1.8%
	Not applicable	6	1.5%

Resource	Response	n	Percent
Scratching opportunities	More than one	180	45.8%
	One	200	50.9%
	Unsure/don't know	5	1.3%
	Not applicable	8	2.0%
A water bowl	More than one	88	22.4%
	One	297	75.6%
	Unsure/don't know	4	1.0%
	Not applicable	4	1.0%
A food bowl	More than one	191	48.6%
	One	193	49.1%
	Unsure/don't know	5	1.3%
	Not applicable	4	1.0%

Note: Percentages are based on 392, 393, 389, 385, 390, 392, 372, and 393 non-missing responses respectively.

Q26. When providing this item in a cat's pen/unit, how many would typically be provided for cats housed together?

Resource	Response	n	Percent
A hiding place at ground level	More than one per cat	186	47.4%
	One per cat	143	36.5%
	Less than one per cat	32	8.2%
	Unsure/don't know	9	2.3%
	Not applicable	22	5.6%
An elevated surface such as a shelf	More than one per cat	184	47.3%
	One per cat	123	31.6%
	Less than one per cat	59	15.2%

Resource	Response	n	Percent
	Unsure/don't know	8	2.1%
	Not applicable	15	3.9%
A hiding place on an elevated area such as a shelf	More than one per cat	150	39.0%
	One per cat	145	37.7%
	Less than one per cat	60	15.6%
	Unsure/don't know	11	2.9%
	Not applicable	19	4.9%
A litter tray that is located away from beds, food and water bowls	More than one per cat	207	53.1%
	One per cat	150	38.5%
	Less than one per cat	13	3.3%
	Unsure/don't know	5	1.3%
	Not applicable	15	3.8%
An area with soft material such as a blanket or cat bed	More than one per cat	256	65.3%
	One per cat	105	26.8%
	Less than one per cat	10	2.6%
	Unsure/don't know	7	1.8%
	Not applicable	14	3.6%
Puzzle feeding devices	More than one per cat	96	25.8%
	One per cat	152	40.9%
	Less than one per cat	49	13.2%
	Unsure/don't know	37	9.9%
	Not applicable	38	10.2%
Toys	More than one per cat	342	87.0%

Resource	Response	n	Percent
	One per cat	22	5.6%
	Less than one per cat	4	1.0%
	Unsure/don't know	9	2.3%
	Not applicable	16	4.1%
Scratching opportunities	More than one per cat	160	40.7%
	One per cat	126	32.1%
	Less than one per cat	77	19.6%
	Unsure/don't know	11	2.8%
	Not applicable	19	4.8%
A water bowl	More than one per cat	109	27.7%
	One per cat	195	49.6%
	Less than one per cat	69	17.6%
	Unsure/don't know	6	1.5%
	Not applicable	14	3.6%
A food bowl	More than one per cat	175	44.5%
	One per cat	190	48.3%
	Less than one per cat	6	1.5%
	Unsure/don't know	8	2.0%
	Not applicable	14	3.6%

Note: Percentages are based on 392, 389, 385, 390, 392, 372, 393, 393, 393, and 393 non-missing responses respectively.

Q45. For resident healthy adult cats (i.e. disease free), how often would:

Cleaning practices	Response	n	Percent
The cat's pen be spot cleaned (i.e. only soiled items removed/dirty surfaces cleaned)	Once a month or less	4	1.11%
	A few times a month	2	0.55%
	Once or twice a week	11	3.05%
	Every day or most days	337	93.35%
	Unsure/don't know	7	1.94%
All/most vertical and horizontal surfaces within a cat’s pen be fully cleaned with disinfectant	Once a month or less	61	16.90%
	A few times a month	52	14.40%
	Once or twice a week	140	38.78%
	Every day or most days	77	21.33%
	Unsure/don't know	31	8.59%
Any non-soiled soft furnishings (i.e. beds, blankets, towels) be removed and replaced with clean items	Once a month or less	58	16.07%
	A few times a month	83	22.99%
	Once or twice a week	154	42.66%
	Every day or most days	44	12.19%
	Unsure/don't know	22	6.09%
Existing litter tray swapped for a clean tray and fresh litter	Once a month or less	14	3.88%
	A few times a month	14	3.88%
	Once or twice a week	103	28.53%
	Every day or most days	229	63.43%
	Unsure/don't know	1	0.28%

Cleaning practices	Response	n	Percent
Fresh food be provided in a clean bowl/tray and/or puzzle feeding devices	Once a month or less	0	0.0%
	A few times a month	0	0.0%
	Once or twice a week	4	1.11%
	Every day or most days	357	98.89%
	Unsure/don't know	0	0.0%
Fresh water be provided in a clean bowl	Once a month or less	0	0.0%
	A few times a month	0	0.0%
	Once or twice a week	9	2.49%
	Every day or most days	352	97.51%
	Unsure/don't know	0	0.0%

Note: Percentages are based on 361 non-missing responses.

Q46. When undertaking cleaning of pens/units/rooms/areas occupied by healthy adult cats, how often would you:

Handling practices	Response	n	Percent
Physically move the cat from where they are currently located in their pen to another area of their pen to facilitate cleaning	Never	82	22.71%
	Sometimes or occasionally	148	41.00%
	Usually	68	18.84%
	Always	40	11.08%
	Unsure/don't know	23	6.37%
Physically place the cat into a carrier or another area outside of their pen to facilitate cleaning	Never	121	33.52%

Handling practices	Response	n	Percent
	Sometimes or occasionally	182	50.42%
	Usually	17	4.71%
	Always	18	4.99%
	Unsure/don't know	23	6.37%
Leave or avoid cleaning certain areas so as not to disturb a cat (i.e. that is hiding/resting/sleeping)	Never	25	6.93%
	Sometimes or occasionally	183	50.69%
	Usually	82	22.71%
	Always	47	13.02%
	Unsure/don't know	24	6.65%
Use separate gloves in between pens	Never	17	4.71%
	Sometimes or occasionally	65	18.01%
	Usually	39	10.80%
	Always	209	57.89%
	Unsure/don't know	31	8.59%
Wash or sanitise hands in between pens	Never	6	1.66%
	Sometimes or occasionally	35	9.70%
	Usually	37	10.25%
	Always	262	72.58%
	Unsure/don't know	21	5.82%
Use separate protective clothing items (e.g. any or combination of aprons,	Never	38	10.53%

Handling practices	Response	n	Percent
overshoes, overalls or body suits) in between pens			
	Sometimes or occasionally	114	31.58%
	Usually	41	11.36%
	Always	137	37.95%
	Unsure/don't know	31	8.59%
Use separate pen cleaning items/equipment in between pens	Never	15	4.16%
	Sometimes or occasionally	45	12.47%
	Usually	48	13.30%
	Always	224	62.05%
	Unsure/don't know	29	8.03%
Remove any soiled soft furnishings (i.e. beds, blankets, towels) and replace with clean items	Never	0	0.00%
	Sometimes or occasionally	1	0.28%
	Usually	11	3.05%
	Always	342	94.74%
	Unsure/don't know	7	1.94%
Removed any soiled litter from trays (and replace with fresh litter where needed)	Never	0	0.00%
	Sometimes or occasionally	0	0.00%
	Usually	8	2.22%
	Always	349	96.68%
	Unsure/don't know	4	1.11%

Handling practices	Response	n	Percent
Clean litter trays away from food preparation areas or at times outside of food preparation times	Never	5	1.39%
	Sometimes or occasionally	4	1.11%
	Usually	15	4.16%
	Always	329	91.14%
	Unsure/don't know	8	2.22%
Fully clean, disinfect and dry out a pen and any items within it at change of occupancy	Never	0	0.00%
	Sometimes or occasionally	4	1.11%
	Usually	7	1.94%
	Always	340	94.18%
	Unsure/don't know	10	2.77%

Note: Percentages are based on 361 non-missing responses.

Q35. Are cat's parasite treatments kept up to date?

Response	n	Percent
Never	0	0.0%
Sometimes or occasionally	2	0.54%
Usually	24	6.52%
Always	338	91.85%
Unsure/don't know	4	1.09%

Note: Percentages are based on 368 non-missing responses.

Q36. Are cat's vaccination courses kept up to date?

Response	n	Percent
Never	3	0.82%
Sometimes or occasionally	12	3.26%
Usually	21	5.71%
Always	331	89.95%
Unsure/don't know	1	0.27%

Note: Percentages are based on 368 non-missing responses.

Q37. Are cats seen by a registered Veterinary Surgeon (RCVS) or Veterinary Nurse (RVN) for a routine health check/screening prior to homing?

Response	n	Percent
Never	3	0.82%
Sometimes or occasionally	1	0.27%
Usually	14	3.80%
Always	348	94.57%
Unsure/don't know	2	0.54%

Note: Percentages are based on 368 non-missing responses.

Q38b. What is the average time from a cat's arrival to them being seen by a vet or vet nurse for a routine health check/screening? If you don’t know the exact number, please estimate.

Response	n	Percent
1	17	6.69%
2	59	23.23%
3	64	25.20%
4	28	11.02%

Response	n	Percent
5	38	14.96%
6	2	0.79%
7	35	13.78%
10	5	1.97%
14	5	1.97%
21	1	0.39%

Note: Percentages are based on 254 non-missing responses.

Q44. During any routine health checks cats experience (i.e. those performed by either cat care staff or vets/nurses), how often are the following methods applied:

Method	Response	n	Percent
Cat restrained by using minimal touch or lightly holding the cat's body/head still with person's arms/hands	Never	2	0.58%
	Sometimes or occasionally	66	19.02%
	Usually	194	55.91%
	Always	64	18.44%
	Unsure/don't know	21	6.05%
Cat restrained by firmly holding the cat's body/head still with person's arms/hands	Never	28	8.07%
	Sometimes or occasionally	243	70.03%
	Usually	37	10.66%
	Always	12	3.46%
	Unsure/don't know	27	7.78%
Cat restrained using a towel wrapped around them	Never	41	11.82%
	Sometimes or occasionally	254	73.20%

Method	Response	n	Percent
	Usually	18	5.19%
	Always	4	1.15%
	Unsure/don't know	30	8.65%
Treats used to get the cat to remain still/to move into a desired position	Never	43	12.39%
	Sometimes or occasionally	185	53.31%
	Usually	69	19.88%
	Always	20	5.76%
	Unsure/don't know	30	8.65%
Cat restrained using a sedative	Never	119	34.29%
	Sometimes or occasionally	180	51.87%
	Usually	2	0.58%
	Always	1	0.29%
	Unsure/don't know	45	12.97%
Cat restrained using a carrier/cage with sliding sections that restrict the cat's movement	Never	88	25.36%
	Sometimes or occasionally	219	63.11%
	Usually	6	1.73%
	Always	2	0.58%
	Unsure/don't know	32	9.22%
Cat restrained by scruffing or applying a clip to the back of their neck	Never	261	75.22%
	Sometimes or occasionally	49	14.12%
	Usually	0	0.00%
	Always	0	0.00%

Method	Response	n	Percent
	Unsure/don't know	37	10.66%
Cat restrained by using a thundershirt, muzzle, headcollar or similar equipment designed to restrict elements of the cat’s movement or behaviour	Never	290	83.57%
	Sometimes or occasionally	17	4.90%
	Usually	0	0.00%
	Always	0	0.00%
	Unsure/don't know	40	11.53%

Note: Percentages are based on 347 non-missing responses.

Q81. Do you hold any formal qualifications in animal behaviour, welfare, health or animal training?

Response	n	Percent
Yes	135	34.4%
No	235	59.8%
Prefer not to say	23	5.9%

Note: Percentages are based on 393 non-missing responses.

Q81. Please specify your formal qualifications in animal behaviour, welfare, health or animal training

Response	n	Percent
Masters/level 7 qualification	11	10.4%
BSc/level 6 qualification	32	30.2%
Level 5 qualification	3	2.8%
Level 4 qualification	4	3.8%
Level 3 qualification	18	17.0%
Level 2 qualification	14	13.2%

Diploma at unknown level	15	14.2%
RVN (qualification unknown)	9	8.5%

Note: Percentages are based on 106 non-missing affirmative responses to the question: "Do you hold any formal qualifications in animal behaviour, welfare, health or animal training?"

Q6. Have you undertaken any of the following types of training in support of your current role?

Training	Response	n	Percent
Understanding and meeting cat's basic needs within the shelter environment	Yes	351	89.31%
	No	42	10.69%
Recognising good and also poor welfare in cats in the shelter environment	Yes	318	80.92%
	No	75	19.08%
Understanding and interpreting cat's behaviour and body language	Yes	344	87.53%
	No	49	12.47%
Interacting with and handling cats appropriately	Yes	337	85.75%
	No	56	14.25%
Disease prevention, recognition and outbreak control	Yes	325	82.70%
	No	68	17.30%
Pen and associated equipment cleaning	Yes	312	79.39%
	No	81	20.61%
Unsure/can't remember		2	0.51%
Other		55	29.73%
No training undertaken		21	5.34%

Q7. When did you last receive any of this type of training?

Training	Time-Frame	n	Percent
Understanding and meeting cat's basic needs within the shelter environment	Within the last 12 months	182	51.85%
	1-2 years ago	61	17.38%
	2-3 years ago	25	7.12%
	3-4 years ago	26	7.41%
	4-5 years ago	16	4.56%
	More than 5 years ago	24	6.84%
	Unsure/can't remember	17	4.84%
Recognising good and also poor welfare in cats in the shelter environment	Within the last 12 months	164	51.57%
	1-2 years ago	68	21.38%
	2-3 years ago	22	6.92%
	3-4 years ago	17	5.35%
	4-5 years ago	6	1.89%
	More than 5 years ago	16	5.03%
	Unsure/can't remember	25	7.86%
Understanding and interpreting cat's behaviour and body language	Within the last 12 months	177	51.45%
	1-2 years ago	61	17.73%
	2-3 years ago	30	8.72%
	3-4 years ago	18	5.23%
	4-5 years ago	20	5.81%
	More than 5 years ago	18	5.23%
	Unsure/can't remember	20	5.81%

Training	Time-Frame	n	Percent
Interacting with and handling cats appropriately	Within the last 12 months	159	47.18%
	1-2 years ago	58	17.21%
	2-3 years ago	30	8.90%
	3-4 years ago	31	9.20%
	4-5 years ago	16	4.75%
	More than 5 years ago	25	7.42%
	Unsure/can't remember	18	5.34%
Disease prevention, recognition and outbreak control	Within the last 12 months	173	53.23%
	1-2 years ago	53	16.31%
	2-3 years ago	35	10.77%
	3-4 years ago	19	5.85%
	4-5 years ago	9	2.77%
	More than 5 years ago	19	5.85%
	Unsure/can't remember	17	5.23%
Pen and associated equipment cleaning	Within the last 12 months	148	47.44%
	1-2 years ago	63	20.19%
	2-3 years ago	26	8.33%
	3-4 years ago	22	7.05%
	4-5 years ago	9	2.88%
	More than 5 years ago	28	8.97%
	Unsure/can't remember	16	5.13%

Note: Percentages are based on 312 - 351 non-missing responses to the question: "Have you undertaken any of the following types of training in support of your current role?"

Q4. Do you currently provide or have you previously provided foster care for cats within your private residence whilst working or volunteering for your current charity/organisation?

Response	n	Percent
Yes	240	61.1%
No	153	38.9%

Note: Percentages are based on 393 non-missing responses.

Q5. Were your premises inspected (either in person or virtually) and assessed before you were able to foster cats?

Response	n	Percent
Yes	163	67.9%
No	67	27.9%
Unsure/don’t know/can’t remember	10	4.17%

Note: Percentages are based on 240 affirmative responses to the question: “Do you currently provide or have you previously provided foster care for cats within your private residence whilst working or volunteering for your current charity/organization?”.

Q80. How many of your *own cats* currently live with you?

Response	Number of kittens/cats	n	Percent
Kittens ages aged 16 weeks or under	0	302	76.8%
	1	5	1.3%
	2	1	0.3%
Cats over 16 weeks of age	0	2	0.5%
	1	116	29.5%
	2	77	19.6%
	3	38	9.7%
	4	19	4.8%
	5	15	3.8%

	6	12	3.1%
	7	10	2.5%
	8	6	1.5%
	9	6	1.5%
	10	2	0.5%
	12	2	0.5%
	14	2	0.5%
	21	1	0.3%
I don’t currently live with any cats or kittens		85	21.6%

Note: Percentages are based on 393 non-missing responses.

Q15. How many cats (including any kittens) on average would a single person be responsible for caring for on a typical day?

Response	n	Percent
1 - 10	172	43.77%
11 - 20	93	23.66%
21 - 30	26	6.62%
31 - 40	10	2.54%
41 - 50	2	0.51%
61 - 70	1	0.25%
Unsure/don’t know	89	22.65%

Note: Percentages are based on 393 non-missing responses.

Q2. How many hours a week on average do you work or volunteer for your charity/organisation?

Role	Weekly Hours	n	Percent
Paid	1 - 9	1	0.6%

Role	Weekly Hours	n	Percent
	9 - 17	3	1.9%
	17 - 25	14	8.7%
	25 - 33	18	11.2%
	33 - 41	104	64.6%
	41 - 49	13	8.1%
	49 - 57	5	3.1%
	57 - 65	1	0.6%
	65 - 73	2	1.2%
Voluntary	1 - 9	68	34.5%
	9 - 17	46	23.4%
	17 - 25	19	9.6%
	25 - 33	32	16.2%
	33 - 41	15	7.6%
	41 - 49	4	2.0%
	49 - 57	6	3.0%
	57 - 65	3	1.5%
	65 - 73	1	0.5%
	81 – 89	1	0.5%
	89 - 97	2	1.0%
Other	1 - 9	1	11.1%
	9 - 17	0	0.0%
	17 - 25	1	11.1%
	25 - 33	0	0.0%
	33 - 41	3	33.3%
	41 - 49	0	0.0%

Role	Weekly Hours	n	Percent
	49 - 57	0	0.0%
	57 - 65	0	0.0%
	65 - 73	2	22.2%
	81 – 89	1	11.1%
	89 - 97	1	11.1%

Note: Percentages are based on 367 non-missing responses. Reported weekly hours > 90 were removed (n = 6).

Q19. Is a waiting list used to manage enquiries for cats to be admitted/brought into care?

Response	n	Percent
Yes	297	88.13%
No	29	8.61%
Unsure/don't know	11	3.26%

Note: Percentages are based on 337 non-missing responses.

Q22. Are any assessment methods or criteria used to determine the *priority level* of a cat to be admitted/brought into care?

Response	n	Percent
Yes	273	81.01%
No	14	4.15%
Unsure/don't know	50	14.84%

Note: Percentages are based on 337 non-missing responses.

Q21. Are any assessment methods or criteria used to determine whether a cat is suitable to admit/bring into care or not?

Response	n	Percent
Yes	235	69.73%

Response	n	Percent
No	44	13.06%
Unsure/don't know	58	17.21%

Note: Percentages are based on 337 non-missing responses.

Q18. How many separate units/pens/rooms/areas are there to house cats? If you don't know the exact number, please estimate.

Response	n	Percent
1 - 10	110	37.80%
11 - 20	51	17.53%
21 - 30	34	11.68%
31 - 40	28	9.62%
41 - 50	28	9.62%
51 - 60	11	3.78%
61 - 70	8	2.75%
71 - 80	8	2.75%
81 - 90	3	1.03%
91 - 100	6	2.06%
101 - 110	1	0.34%
111 - 120	1	0.34%
121 - 130	1	0.34%
141 - 150	1	0.34%

Note: Percentages are based on 291 non-missing responses.

Q20. How many cats are currently on the waiting list? If you don't know the exact number, please estimate.

Response	n	Percent
0 - 25	98	44.75%
25 - 50	43	19.63%
50 - 75	19	8.68%
75 - 100	9	4.11%
100 - 125	18	8.22%
125 - 150	5	2.28%
150 - 175	11	5.02%
175 - 200	2	0.91%
200 - 225	3	1.37%
225 - 250	3	1.37%
250 - 275	1	0.46%
275 - 300	1	0.46%
300 - 325	3	1.37%
325 - 350	1	0.46%
375 - 400	1	0.46%
450 - 475	1	0.46%

Note: Percentages are based on 219 non-missing responses.

Q16. How many cats are there in care at the moment? *If you don't know the exact number, please estimate.*

Response	n	Percent
0 - 10	72	22.36%
10 - 20	47	14.60%
20 - 30	53	16.46%
30 - 40	28	8.70%

Response	n	Percent
40 - 50	33	10.25%
50 - 60	24	7.45%
60 - 70	19	5.90%
70 - 80	14	4.35%
80 - 90	8	2.48%
90 - 100	4	1.24%
100 - 110	9	2.80%
110 - 120	1	0.31%
120 - 130	2	0.62%
140 - 150	3	0.93%
150 - 160	1	0.31%
160 - 170	1	0.31%
180 - 190	2	0.62%
200 - 210	1	0.31%

Note: Percentages are based on 322 non-missing responses.

Q23. Are there units/pens/areas that are routinely kept empty and reserved for emergency admission/intake situations?

Response	n	Percent
Never	61	18.1%
Sometimes or occasionally	124	36.8%
Usually	71	21.1%
Always	53	15.7%
Unsure/don't know	28	8.3%

Note: Percentages are based on 337 non-missing responses.

Q41. If cats have a suspected or confirmed contagious disease, are there options for them to be placed in isolation facilities? (i.e. kept in a self-contained pen/unit/room/area away from healthy cat populations)

Response	n	Percent
Yes	328	89.13%
No	25	6.79%
Unsure/don't know	15	4.08%

Note: Percentages are based on 368 non-missing responses.

Q18. Over the past 12 months what is the average time from a cat entering your site/cattery/centre to it leaving?

Cat type/outcome	Days range	n	Percent
TNR/R	0 - 9	106	51.46%
	10 - 19	13	6.31%
	20 - 29	8	3.88%
	30 - 39	1	0.49%
	40 - 49	4	1.94%
	50 - 59	2	0.97%
	60 - 69	1	0.49%
	90 - 99	2	0.97%
	Unsure/don’t know	69	33.50%
Rehomed	1 - 10	14	4.22%
	11 - 20	44	13.25%
	21 - 30	86	25.90%
	31 - 40	33	9.94%
	41 - 50	27	8.13%
	51 - 60	22	6.63%

Cat type/outcome	Days range	n	Percent
	61 - 70	6	1.81%
	71 - 80	1	0.30%
	81 - 90	23	6.93%
	91 - 100	4	1.20%
	111 - 120	3	0.90%
	141 - 150	1	0.30%
	151 - 160	1	0.30%
	171 - 180	4	1.20%
	191 - 200	2	0.60%
	261 - 270	1	0.30%
	361 - 370	1	0.30%
	521 - 530	1	0.30%
	Unsure/don’t know	58	17.47%

Note: Percentages are based on 206 and 332 non-missing responses respectively.

Q18. Over the past 12 months what is the shortest time from a cat entering your site/cattery/centre to it leaving?

Cat type/outcome	Days range	n	Percent
TNR/R	0 - 9	126	63.32%
	10 - 19	8	4.02%
	20 - 29	5	2.51%
	50 - 59	1	0.50%
	60 - 69	1	0.50%
	Unsure/don’t know	58	29.15%
Rehomed	0 - 9	164	49.25%
	10 - 19	52	15.62%

Cat type/outcome	Days range	n	Percent
	20 - 29	36	10.81%
	30 - 39	13	3.90%
	40 - 49	3	0.90%
	60 - 69	4	1.20%
	80 - 89	1	0.30%
	90 - 99	3	0.90%
	200 - 209	1	0.30%
	250 - 259	1	0.30%
	Unsure/don't know	55	16.52%

Note: Percentages are based on 199 and 333 non-missing responses respectively.

Q18. Over the past 12 months what is the longest time from a cat entering your site/cattery/centre to it leaving?

Cat type/outcome	Days range	n	Percent
TNR/R	0 - 9	85	42.93%
	10 - 19	16	8.08%
	20 - 29	4	2.02%
	30 - 39	7	3.54%
	40 - 49	6	3.03%
	50 - 59	1	0.51%
	60 - 69	6	3.03%
	70 - 79	1	0.51%
	100 - 109	1	0.51%
	120 - 129	1	0.51%
	150 - 159	1	0.51%
	160 - 169	2	1.01%

Cat type/outcome	Days range	n	Percent
	180 - 189	2	1.01%
	230 - 239	1	0.51%
	360 - 369	1	0.51%
	Unsure/don't know	63	31.82%
Rehomed	0 - 9	4	1.20%
	10 - 19	3	0.90%
	20 - 29	8	2.40%
	30 - 39	6	1.80%
	40 - 49	13	3.90%
	50 - 59	2	0.60%
	60 - 69	13	3.90%
	70 - 79	4	1.20%
	80 - 89	8	2.40%
	90 - 99	20	6.01%
	100 - 109	11	3.30%
	110 - 119	7	2.10%
	120 - 129	12	3.60%
	130 - 139	4	1.20%
	140 - 149	7	2.10%
	150 - 159	20	6.01%
	160 - 169	11	3.30%
	170 - 179	2	0.60%
	180 - 189	24	7.21%
	190 - 199	5	1.50%
	200 - 209	12	3.60%

Cat type/outcome	Days range	n	Percent
	210 - 219	7	2.10%
	220 - 229	4	1.20%
	230 - 239	1	0.30%
	240 - 249	2	0.60%
	250 - 259	4	1.20%
	260 - 269	1	0.30%
	270 - 279	1	0.30%
	280 - 289	2	0.60%
	300 - 309	7	2.10%
	310 - 319	1	0.30%
	320 - 329	1	0.30%
	330 - 339	2	0.60%
	350 - 359	3	0.90%
	360 - 369	22	6.61%
	370 - 379	3	0.90%
	400 - 409	3	0.90%
	500 - 509	3	0.90%
	510 - 519	1	0.30%
	540 - 549	1	0.30%
	610 - 619	1	0.30%
	860 - 869	1	0.30%
	Unsure/don’t know	66	19.82%

Note: Percentages are based on 198 and 333 non-missing responses respectively.

Q78. Please indicate your age

Response	n	Percent
18-24	23	5.9%
25-34	78	19.8%
35-44	68	17.3%
45-54	72	18.3%
55-64	87	22.1%
65-74	52	13.2%
75-84	5	1.3%
85+	1	0.3%
Prefer not to say	7	1.8%

Note: Percentages are based on 393 non-missing responses.

Q 79. Please indicate the number of active years in total you have either worked and/or volunteered within the rehoming/shelter/rescue sector

Response	n	Percent
1	41	10.57%
2	27	6.96%
3	19	4.90%
4	23	5.93%
5	25	6.44%
6	19	4.90%
7	20	5.15%
8	24	6.19%
9	10	2.58%
10	20	5.15%
11	6	1.55%
12	17	4.38%

Response	n	Percent
13	8	2.06%
14	12	3.09%
15	17	4.38%
16	6	1.55%
17	2	0.52%
18	8	2.06%
19	1	0.26%
20	13	3.35%
21	3	0.77%
22	6	1.55%
23	7	1.80%
24	4	1.03%
25	14	3.61%
26	2	0.52%
28	3	0.77%
29	1	0.26%
30	11	2.84%
32	1	0.26%
34	1	0.26%
35	6	1.55%
36	2	0.52%
37	1	0.26%
40	4	1.03%
42	1	0.26%
43	1	0.26%

Response	n	Percent
44	1	0.26%
45	1	0.26%

Note: Percentages are based on 388 non-missing responses.
