# Supplementary material for: Part 1: A Sector-Wide Survey of UK/British Isles Shelter Organisations Caring for Cats: Caregiver-Reported Approaches to Housing, Husbandry and General Care Provision
Source: Vet Sci. 2026 Jun 16;13(6):587. doi: 10.3390/vetsci13060587 (PMC13308460; doi:10.3390/vetsci13060587)
Supplement: Supplementary file 1 [file vetsci-13-00587-s001.zip › Document S3 - ADCH minimum requirements.pdf]

**3.6 Meeting cat's basic needs**		
**Survey sub section:**	**Relevant ADCH minimum requirements:**	**Evidence of relevant ADCH minimum standard met**
3.6.1 Group versus single housing	• *"Only compatible cats shall be housed together. Decisions to home cats together shall be made by a competent person."*<br>• *"Cats from the same household may be compatible and may benefit from each other's company."*<br>• *"Putting together unrelated or incompatible cats can put pet cats under pressure and cause stress."*<br>• *"Single housing is the best choice for cats unless they have been living together harmoniously in a home previously."*<br>• *"….even cats from the same household may be incompatible and should be monitored for signs of fear, stress or persistent aggression. In the event of these instances the cats should be separated either into individual housing, or into amicable groups, dependent on the individual case."*	Evidence minimum standards partially met by majority; more than 50% of participants reported consistently avoiding housing unfamiliar cats together and consistently separating cats not getting on well together. However less than 50% of respondents reported consistently using previous social history of cats to inform social housing decision making
3.6.2 Pen dimensions	• *"The height of cat sleeping areas should be between 75cm and 1.5m. Units should be provided with a sleeping area of 0.85 sq. m for one cat, 1.1 sq. m for two cats, and 1.7 sq. m for up to four cats."*<br>• *"Cat exercise areas should be 1.65 m^2 for single cats; 2.2 m^2 for two cats and 2.8 m^2 for up to 4 cats. The exercise area should be a minimum of 1.8m tall."*<br>• *"The floor area should be equal to the sum of the above sleeping and exercise areas" [see following calculations for rough overall volume]*	Evidence minimum standards met by majority for single and pair housed cats; calculated median volumes reported in the survey were greater than those based on ADCH recommended dimensions. ADCH guidelines do not provide any recommended values for cat groups larger than four, therefore our results regarding volumes for cats housed in groups of 3 or more cannot be directly compared to ADCH guidance.

	*1 cat: combined sleep and exercise area (2.5 m^2) * 1.8 m height = 4.5 m^3*<br><br>*2 cats: combined sleep and exercise area (3.3 m^2) * 1.8 m height = 5.94 m^3*<br>*Up to 4 cats: combined sleep and exercise area (4.5 m^2) * 1.8 m height = 8.1 m^3*	
3.6.3 Resource provisions	• *"Cats shall be provided with a place to hide which shall be large enough to provide concealment"*<br>• *"…cats shall be given facilities for scratching.*<br>• *"All cats shall have toys and/or feeding enrichment to provide opportunities to perform natural behaviour such as hunting or play. Feeding enrichment can be provided by scatter feeding of dried food or biscuits or food filled play balls or other items can be provided to supplement the normal feeding regime."*<br>• *"In cat units, suitably sited and sized litter trays, which are easy to clean and impermeable, shall be provided at all times. They shall be placed as far away as possible from the resting and feeding areas".*<br>• *"Shelves or raised areas shall be provided to allow cats to rest high up."*<br>• *"Suitable bedding shall be provided which allows the animal to be comfortable."*<br>• *"Clean, potable (fit for human consumption) water shall be available at all times."*<br>• *"Pair housed or group-housed cats shall have sufficient space and adequate resources to*	Evidence minimum standards partially met by majority; more than 50% of respondents consistently provided basic resources, with the exception of feeding enrichment. Over 50% of respondents reported providing one or more than one of each resource per cat where multi-housed.

	minimise competition and to be able to avoid each other and hide away separately from one another if they choose. This includes provision of a separate litter tray, bed and hiding place for each cat".	
3.6.4 Biosecurity and cleaning practices in pens occupied by healthy adult cats	• *Eating vessels, which shall be capable of being easily cleansed and disinfected, shall be cleansed or disposed of after each meal and drinking vessels shall be cleaned at least once a day."*<br>• *"Each occupied unit shall be cleaned daily. All excreta and soiled material shall be removed from all areas used by the animals at least daily and more often if necessary.*<br>• *Bedding shall be sited out of draughts and shall be checked daily and maintained in a clean, parasite free and dry condition*<br>• *Litter trays shall be scooped at least once a day and at any time during the day if found to be unduly soiled. Cleaning shall take place away from food preparation areas or at least at separate times.*<br>• *Each unit shall be thoroughly cleaned, disinfected and dried at a change of occupancy".*	Evidence minimum standards met by majority; over 50% of respondents reported providing clean food and water bowls daily, cleaning pens daily or most days, consistently removing soiled items and litter during cleaning, fully cleaning, disinfecting and drying and pens at change of occupancy and cleaning trays away from food preparation areas.
3.6.5 Managing psychological distress during cleaning and husbandry practices	*"Although maintaining a hygienic environment is important, consideration to the comfort and mental welfare of the animals must also be made. Cleaning and husbandry practices need to take into consideration the psychological distress that may be caused by the daily removal of familiar smells and bedding through the use of large amounts of water and disinfectant. Animals can be adversely affected by strong chemical smells, or a constantly damp environment. Housing or bedding that is too rigorously cleaned may remove valuable*	Evidence minimum standards partially met by majority; over 50% of respondents reported regularly spot cleaning however over 50% also reported regular replacement of non-soiled soft furnishings and regular/semi regular full disinfection of pens as well as direct handling and disturbance of cat during cleaning

	scent marking. The use of a disinfectant-led spot cleaning approach satisfies the demands of both welfare and hygiene once the animal has gone through the quarantine period."	
**3.6.6** Preventative health care and health checks prior to homing	• *"Precautions, as advised by the veterinary surgeon, shall be taken to prevent and control the spread of infectious disease and parasites amongst the animals, staff, volunteers and visitors".*<br>• *"Animals shall be vaccinated on or shortly after arrival into the centre under the direction of the veterinary surgeon. Unless recent evidence of vaccination or vet/behaviourist advises against vaccination".*<br>• *"A health check by a vet or a suitably competent lay person should take place on admission or as close to (no more than 72 hours)".*<br>• *"On being adopted by their new owner, every animal shall have been fully examined before departure, if not by a veterinary surgeon then by a suitably skilled and competent person".*	Evidence minimum standards met by majority; over 50% of respondents reported parasite treatments and vaccinations kept up to date and over 50% of respondents reported cats consistently seen by a vet prior to homing and either on the first day or within first week of arrival
3.6.7 Cat handling during routine health checks	No standards available for comparison	
**3.8 Local site capacity and operations**		
3.8.1 Caregiver qualifications and role-based training	• *"People must be trained to a level of competence suitable to ensure the care and welfare needs of the animals in their care ….The training policy should be reviewed and updated on an annual basis"*<br>• *"People should hold a relevant qualification and/or be able to clearly evidence knowledge through experience, for example through on the job in-house training (such as shadowing from a qualified*	Evidence minimum requirements met by majority; less than 50% of participants reported holding a relevant formal qualification, howeverer over 50% of participants reported undertaking work-based training on animal-welfare related topics in support of their current role and within the past 12 months. The guidelines do not specify how often training should be undertaken or the specific types of training or qualifications that are recommended.

	and experienced individual)" [No particular qualifications are specified within the requirements, but links are provided to a portal to check qualification legitimacy].	
3.8.2 Caregiver fostering and cat ownership	• *"Each home which is used to provide foster care shall be visited in person or virtually, assessed and approved by a suitably knowledgeable member of the organisation's staff or volunteers, or another ADCH member, before it is used for fostering animals".*<br>• *"An assessment shall be made, and steps in place to avoid the risk of overcrowding and hoarding both owned and foster cats [no maximum numbers of cats specified], and agreement reached with the foster carer".*	Evidence minimum standards partially met by majority; over 50% of participants reported premises inspected prior to fostering, however parameters for determining overcrowding or hoarding risk not specified and therefore cannot be compared against reported cat ownership rates etc.
3.8.3 Caregiver to cat ratios	*"Numeric ratios are not prescribed in these standards, as the individual animals being cared for can require different levels of care depending on their state of physical health, mental health, history and breeding. In calculating the people to animal ratio at any given time, consideration must be foremost given to ensuring that there is enough capacity between all people to be able to provide the five welfare needs for every individual animal onsite. If animals cannot be cared for under the five welfare needs (glossary 4), then animal intake is to be correspondingly reduced"*	Standards do not specify values therefore comparison not possible
3.8.4 Number of hours worked/volunteered per week	No standards available for comparison	
3.8.5 Waiting lists and intake processes	No standards available for comparison	

3.8.6 Proportionate pen occupancy	No standards available for comparison	
3.8.7 Emergency intake	No standards available for comparison	
3.8.8 Isolation facilities	• *"A documented procedure should be held covering the organisation's plans for infectious and potentially infectious animals. This should include agreed locations of isolation pens (if not on site) which should be accessible 24 hours a day".*<br>• *"Adequate isolation facilities for animals with infectious diseases shall be available. This can be at the organisation's own centre, or a formal documented arrangement with a local provider such as a veterinary practice".*	Evidence minimum standards met by majority; over 50% of participants reported there were options for cats to be placed in isolation facilities
3.8.9 Cat length of stay for TNR/R (i.e. 'feral') cats	*"Feral cats must not be kept in confinement any longer than 48 hours, unless immediate veterinary intervention is needed."*	Evidence minimum standards met by majority; median reported 2 day stay for an average 'feral' cat (i.e. those managed under TNR/R pathways)
